# Bioresource Upgrade for Sustainable Energy, Environment, and Biomedicine

**DOI:** 10.1007/s40820-022-00993-4

**Published:** 2023-01-11

**Authors:** Fanghua Li, Yiwei Li, K. S. Novoselov, Feng Liang, Jiashen Meng, Shih-Hsin Ho, Tong Zhao, Hui Zhou, Awais Ahmad, Yinlong Zhu, Liangxing Hu, Dongxiao Ji, Litao Jia, Rui Liu, Seeram Ramakrishna, Xingcai Zhang

**Affiliations:** 1 Center for Nanofibers and Nanotechnology, National University of Singapore, Singapore, 119260 Singapore; 2 State Key Laboratory of Urban Water Resource and Environment, Harbin Institute of Technology, Harbin, 150090 People’s Republic of China; 3 School of Engineering, Massachusetts Institute of Technology, Cambridge, MA 02139 USA; 4 John A Paulson School of Engineering and Applied Sciences, Harvard University, Cambridge, MA 02138 USA; 5 The Key Laboratory for Biomedical Photonics of MOE at Wuhan National Laboratory for Optoelectronics – Department of Biomedical Engineering, College of Life Science and Technology, Huazhong University of Science and Technology, Wuhan, Hubei 430074 People’s Republic of China; 6 Centre for Advanced 2D Materials, National University of Singapore, Singapore, 117546 Singapore; 7 School of Physics and Astronomy, The University of Manchester, Manchester, M13 9PL UK; 8 Department of Anesthesia, Critical Care and Pain Medicine, Massachusetts General Hospital and Harvard Medical School, Boston, MA 02114 USA; 9 Department of Energy and Power Engineering, Tsinghua University, Beijing, 100084 People’s Republic of China; 10 Departamento de Quimica Organica, Universidad de Cordoba, Edificio Marie Curie (C-3), Ctra Nnal IV-A, Km 396, 14014 Cordoba, Spain; 11 Department of Chemical Engineering, Monash University, Clayton, VIC 3800 Australia; 12 School of Mechanical and Aerospace Engineering, Nanyang Technological University, Singapore, 639798 Singapore

**Keywords:** High availability low utilization biomass (HALUB), Circular economy, Machine learning, Energy-efficient conversion, Nano-catalysis

## Abstract

We conceptualize bioresource upgrade for sustainable energy, environment, and biomedicine with a focus on circular economy, sustainability, and carbon neutrality using high availability and low utilization biomass (HALUB). We acme energy-efficient technologies for sustainable energy and material recovery and applications. The technologies of thermochemical conversion (TC), biochemical conversion (BC), electrochemical conversion (EC), and photochemical conversion (PTC) are summarized for HALUB. Microalgal biomass could contribute to a biofuel HHV of 35.72 MJ Kg^−1^ and total benefit of 749 $/ton biomass via TC. Specific surface area of biochar reached 3000 m^2^ g^−1^ via pyrolytic carbonization of waste bean dregs. Lignocellulosic biomass can be effectively converted into bio-stimulants and biofertilizers via BC with a high conversion efficiency of more than 90%. Besides, lignocellulosic biomass can contribute to a current density of 672 mA m^−2^ via EC. Bioresource can be 100% selectively synthesized via electrocatalysis through EC and PTC. Machine learning, techno-economic analysis, and life cycle analysis are essential to various upgrading approaches of HALUB. Sustainable biomaterials, sustainable living materials and technologies for biomedical and multifunctional applications like nano-catalysis, microfluidic and micro/nanomotors beyond are also highlighted. New techniques and systems for the complete conversion and utilization of HALUB for new energy and materials are further discussed.

## Highlights

- Machine learning, techno-economic analysis, and life cycle analysis are imperative for various conversion approaches of high availability and low utilization biomass (HALUB).
- The conversion of HALUB to sustainable energy and materials has a positive consequence on mitigating climate change and building a green future.
- Microfluidic and micro/nanomotors-powered sustainable materials are of high potential for advanced applications.

## Graphical abstract

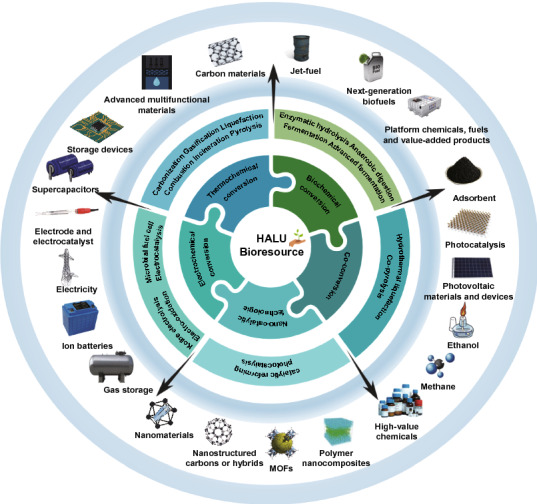

## Introduction

More than 80% of the global energy requirements are gratified by fossil fuels, which manifest the dependency of human beings on non-renewable resources. This review exclusively aims to investigate novel raw materials with high availability and low utilization bioresource (HALUB), which are abundant in nature around the Globe, tell-tale their high potential to achieve carbon neutrality in environment, largely unutilized at a significant level. Among the various renewable biofuels, HALUB is one of the most appealing feedstocks to produce gasoline substitutes or carbon–neutral materials in order to reduce the use of other non-renewable sources, such as petroleum resources and the concentration of CO_2_ [1, 2]. Owing to the environmentally benign and biodegradable nature, the carbon materials derived from bioresources can be used in energy conversion, storage, catalysis, water purification, biomedicine and biotechnology applications [3, 4]. Hence, it is of significant importance to explore this research field and its potential submissions.

Bioresource upgrade is an emerging frontier due to its importance in energy, environmental and biomedical applications. For instance, the lignocellulose can be used as a bioresource in the production of hydrocarbons, for transport and the generation of chemical building blocks for green synthesis, biotechnology, or biomedicine. Prior to the production of bioethanol, the lignocellulosic biomass can also be utilized via both catalytic and non-catalytic degradation [5]. Anaerobic digestion (AD) and microbial fuel cell (MFC) are promising in the process for HALUB conversion into energy [6]. Owing to their high lignin solubility, wide potential to avoid water splitting reaction, the ionic liquids (ILs) have attracted attention as alternative electrolytes. While the high-cost of ILs has limited their commercial use, hydrothermal carbonization (HTC) and hydrothermal liquefaction (HTL) can be used as alternatives to conventional fuels due to the synthesis of high-value-added carbonaceous materials and biofuels [7, 8]. The hydrothermal conversion of food residues and lignocellulosic biomass has been studied via HTC and HTL [9, 10]. Waste yeast biomass-derived materials can help develop eco-friendly catalysts for sustainable water splitting and hydrogen production [11, 12]. Machine learning can be very helpful to predict the performance and mechanism of the HALUB conversion processes and to enhance the conversion efficiency during the process of HTC and HTL [11, 12].

**Fig. 1:**
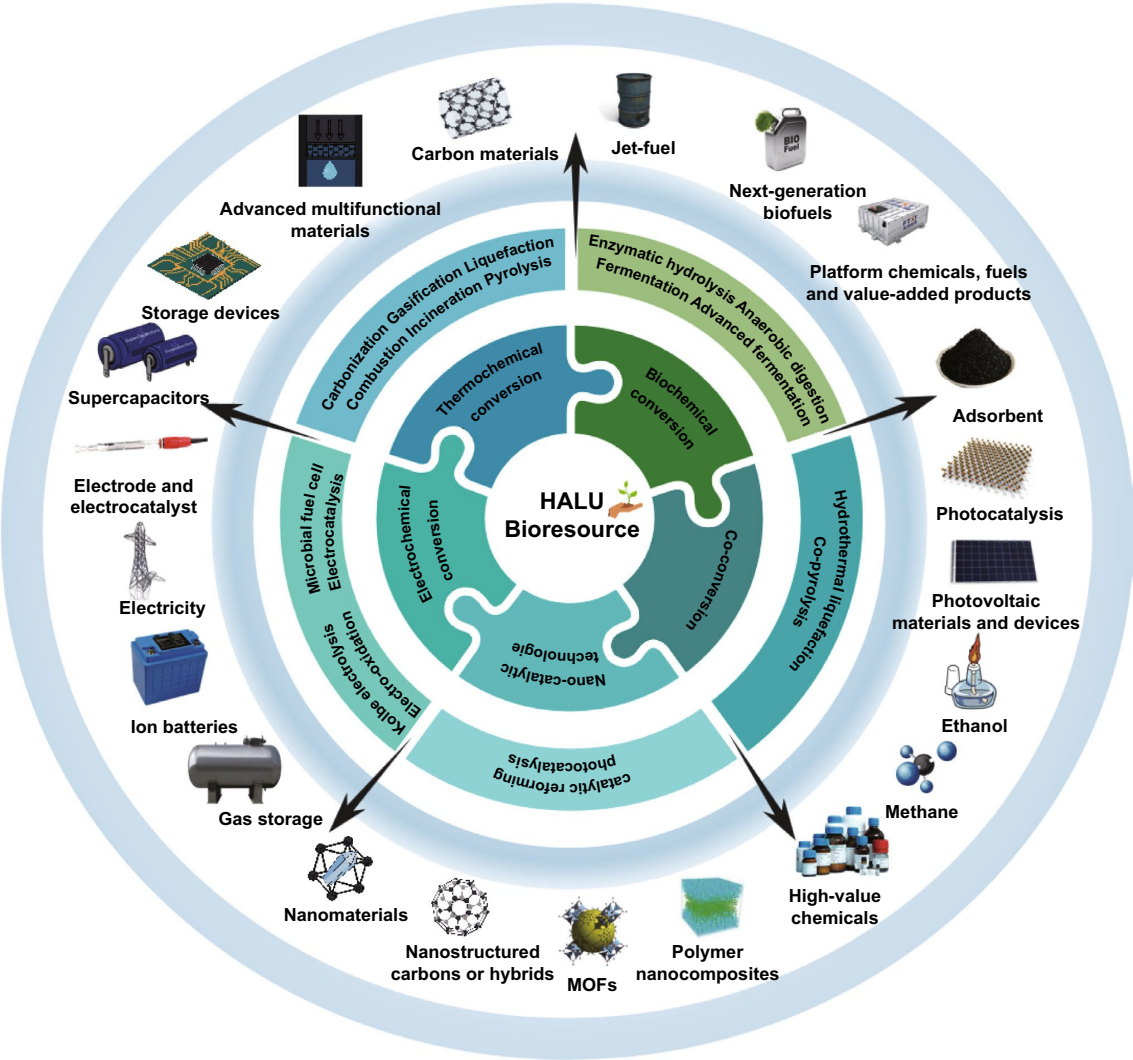
Applications of the HALUB

The effectual conversion of HALUB is very significant in the production of high-value chemicals and the solid waste management [13]. Biochemical conversion can be used to produce green chemicals and biofuels via enzymatic hydrolysis, anaerobic digestion, fermentation, and separation. Furthermore, alkali-based pretreatment occupies its role in the development of ethanol production technologies from cellulosic biomass [13]. The combined biorefineries are of high potential for the cleaner production of biofuels, food, bio-based polymers, bio-based chemicals, and pharmaceuticals [7, 14]. The selection of non-food raw materials, the advanced conversion and management technologies are highly important in the sustainable production of biofuels, as alternatives to biofuel synthesis from energy crops like sorghum and non-staple miscanthus. Most of the HALUB like straw is returned to the soil, while researchers are exploring the pyrolysis, gasification, and other new technologies to convert and manage these biowastes. HALUB can be used to produce electricity, syngas, carbon materials, biofuels and electrolyzer fuel cell, playing a significant role in life science and environmental sustainability, especially to moderate climate change from a long-term perspective. Various applications of the HALUB are summarized in Fig. 1.

The world's total carbon emissions are about 30 billion tons per year [15, 16]. It is exceedingly energy-consuming and cost-ineffective to neutralize these emissions at a large scale. Reproducing energy and materials from the renewable bioresources is an economic approach to neutralize carbon emissions. Furthermore, it is urgent to find other eco-friendly sources for sustainable electrochemical products such as supercapacitors, dye-sensitized solar cells, and sensors.

Here we afford an overview of biochemical, electrochemical, thermochemical conversion, as well as nano-catalytic technologies and machine learning for advanced energy materials. Biochemical conversion (BC) includes enzymatic hydrolysis, anaerobic digestion, fermentation, and advanced fermentation [17]. Electrochemical conversion (EC) comprises valorization of lignin, microbial fuel cells, and fuel cell system [17]. Thermochemical conversion (TC) includes hydrothermal carbonization, pyrolysis, gasification, liquefaction, and combustion [17]. A combination of different conversion technologies is discussed, such as integration of (1) EC and BC, (2) TC and BC and (3) EC, TC and BC.

**Fig. 2:**
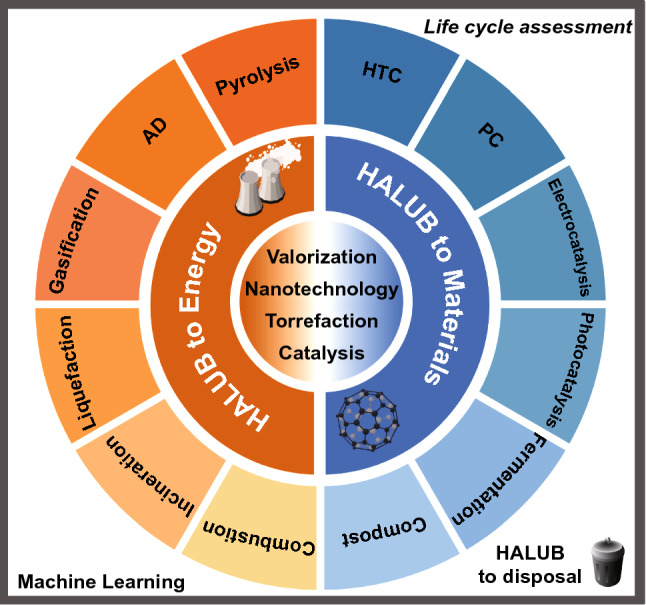
Promising technologies for HALUB conversion to energy and materials

Promising technologies for renovating HALUB to energy and materials are summarized in Fig. 2. Among these technologies, biotechnology, nanotechnology, electrocatalysis and photocatalysis are the most appealing fields in recent years [18]. In terms of novel fuel cells, single-component fuel cells have been reported to address the technical bottlenecks of solid oxide fuel cells which were difficult to commercialize. These advances have urged researchers to sightsee the development of international fuel cells. The CO_2_ released in atmosphere happened when biomass burned for electricity or heat. Although, biomass, like tree, crop as well as agricultural plant captures CO_2_ with photosynthesis process. The carbon-negative property of biomass energy needs attention of researchers and thus the development of the biomass energy industries could be promoted. In total, following sections of the review exclusively demonstrate the relevant information and studies.

## Bioresource Components and Material Properties

### Biomass-Based Material Properties

**Fig. 3:**
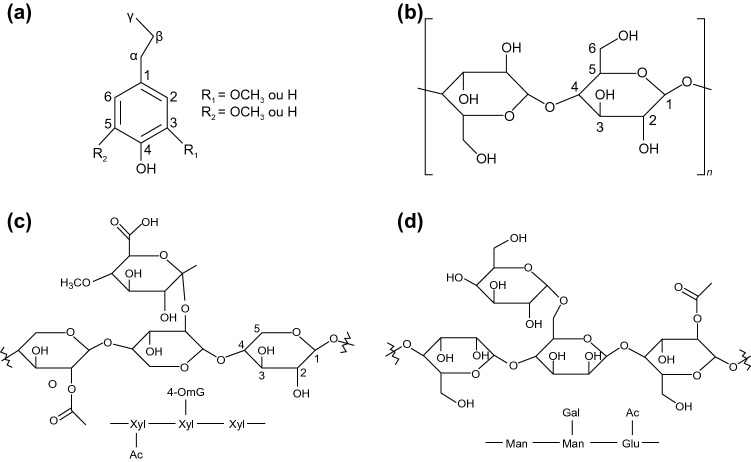
Basic constituents of biomass, **a** lignin, **b** cellulose, **c** xylans, and **d** glucomannan, respectively [27]

Biomass is classified into the following major groups: fungal biomass, bacterial biomass, marine algae and plant biomass, herbaceous and agricultural biomass, human waste biomass, industrial waste or contaminated semi-biomass, and animal biomass [19]. The composition of biomass often depends on its type and growing conditions, and, in addition, for plant-based biomass composition depends to a large extent on the soil in which it grows [20]. However, in most cases, biomass contains cellulose, hemicellulose, lignin, polysaccharides, extracts, lipids, proteins, carbohydrates, water, hydrocarbons, ash and other components [21–26]. The composition of lignin, cellulose and hemicellulose is given in Fig. 3a–d [27]). In terms of constituent elements, biomass can contain a large number of ash-forming elements, i.e., Na, K, Ca, Mg, Si, Ti, Al, and Mg [28], in addition to the major C, H and O and the minor non-metallic N, P and S elements [29]. These elements are essential for the properties of biomass-derived materials due to doping. In addition, trace elements such as Zn, Cu, Mo, Mn, Fe are important in biomass processing and properties of biomass-derived materials because they can act as catalysts for the reactions generating biomass-derived materials [30].

It is noteworthy that the main focus of most reviews and research articles is on biomass processing methods. There is no doubt that the development of efficient biomass processing methods is crucial for the development of the field of biomass-derived materials. However, if the influence of biomass components on the performance of biomass-derived materials can be clarified, blind selection of biomass precursors will be avoided and biomass sources with a certain high percentage of components can be specifically selected according to the application of biomass-derived materials, which should enhance the performance of biomass-derived materials.

Interestingly enough, biomass exhibits a great variability in chemical composition and physical behavior (*O*/*C* and *H*/*C* ratios, particle size structure, ash content). It has been shown that the flammability and explosive behavior of biomass is related to the composition of the biomass, with a greater tendency of spontaneous combustion observed in that biomass with higher *H*/*C* ratios [31]. Furthermore, the development of lignin-rich biomass into nanoparticles (LNP) offers key advantages, such as improved performance of polymer blends and higher antioxidant activity due to a higher surface area to volume ratio [32]. The complex chemical structure of the aromatic ring containing methoxy and hydroxyl groups allows lignin to be incorporated into different materials to produce antioxidant products that can be used in a variety of applications. For example, Lu et al. [33] used a supercritical antisolvent (SAS) method to prepare nanoscale lignin (0.144 ± 0.03 μm) using acetone as the solvent and supercritical carbon dioxide as the antisolvent. The results showed that the SAS process did not lead to degradation of lignin or changes in chemical structure. Due to the increased solubility, many antioxidant parameters of nano-lignin were significantly enhanced, including DPPH radical scavenging activity, superoxide radical scavenging activity and reducing power. As an antioxidant, nanoscale lignin is a better material than non-nanoscale lignin.

Hydrothermal liquefaction is a promising process for producing high-quality bio-oil from biomass. However, different biomass compositions have a significant effect on bio-oil yield. Caprariis et al. [34] compared the effect of three biomass, natural hay, oak and walnut shell, on the yield and quality of bio-oil and found that bio-oil yield increased with increasing lignin content in biomass, with walnut shell having the highest bio-oil yield and cellulose having the lowest bio-oil yield.

Notably, the reconstruction of structural plant components (cellulose, lignin and hemicellulose) into materials with advanced optical properties is a very promising area, as advanced materials made from sustainable resources, including those obtained from industrial or agricultural sidestreams, with potentially lower costs, show great promise in optoelectronics, while meeting or even exceeding current performance requirements. The fabrication of lignocellulose into optical thin films has a wide range of applications. In addition, lignocellulose can be used to produce bio-based UV-blocking materials that can then be used in devices to protect components from the harmful effects of UV light. In addition, bioluminescence can be achieved by integrating lignocellulosic materials with luminescent materials, including lanthanides, carbon quantum dots and other dopants: perovskites, metal halides, and organic dyes [35].

In addition, biomass diffraction materials are widely studied in the field of electrocatalysis. Doping of heteroatoms such as N, B, S, Se, I, and P into carbon nanomaterials can be used as promising electrocatalysts as an alternative to the noble metal Pt. Heteroatoms have different atomic sizes and electronegativity compared to C. Therefore, their doping into graphitic carbon structures leads to changes in their charge distribution and electronic properties. In this regard, N is a special dopant because it is the next neighboring element of C in the periodic table, both of which exhibit similar atomic sizes but different electronegativities. The doping of N into the graphitic carbon structure produces a minimal lattice mismatch. The strong electron-withdrawing ability of the N atom brings a net positive charge to the neighboring C atom through intermolecular charge transfer. Therefore, based on this consideration, biomass precursors with high heteroatom content can be selected in the preparation of biomass diffractive materials for electrocatalysis [19].

Furthermore, the cellulose content is related to the mechanical strength of the biomass, and the structure of cellulose and its crystallinity are other important parameters that affect the mechanical properties. Some authors have observed the influence of the percentage of cellulose on the mechanical properties, and defects or dislocations can be considered as weaknesses of the plant cell wall. Fibers that appear to have the highest levels of cellulose, such as flax or ramie, are those with higher mechanical properties. However, this is not always the case; cotton, despite its high cellulose content, has very low tensile properties. Cellulose rate is not the only reason for the mechanical capacity of plant cell walls [36]. The orientation of the cellulose fibrils relative to the axis is also considered to a factor of the mechanical performance. Different theories, based on the work of mechanical engineers or biologists, enable the arrangements of the different cell wall constituents to be another explanation.

### Biomass-Based Material Synthesis Process

The different components of biomass have an impact on the properties of the derived materials, in addition to the process of material preparation. The processing of biomass into carbon nanomaterials depends to a large extent on the structure of the biomass components. The conversion process of biomass to carbon materials is considered. Selection of the right biomass precursors is a key step in the synthesis of biocarbon materials with the desired structure and morphology. Biomass resources are usually composed of complex components and these chemicals usually have different pyrolysis mechanisms. For example, the decomposition of cellulose in an inert atmosphere typically occurs between 315 and 400 °C [37]. At temperatures above 400 °C, cellulose pyrolyzes and forms a small amount of residual carbon (≈6.5 wt%). As for lignin, the pyrolysis is more complex and can occur over a wider temperature range (140–800 °C), while a larger percentage of residual carbon (41.2 wt%) is produced at 800 ℃ [38].

The decomposition of cellulose, lignin and hemicellulose has different decomposition routes during pyrolytic carbonation. The large number of hydroxyl groups in cellulose and hemicellulose are highly susceptible to degradation to volatile compounds (CO, CO_2_, H_2_O and some hydrocarbons) at low temperatures (≤ 400 °C). At the same time, these processes usually produce many oxygen-containing heterocycles, which are easily converted to aromatic rings by reactions (dehydration, decarboxylation and decarbonylation) as the temperature increases, favoring the formation of BCM with interconnected microstructures. As for lignin, it contains fewer oxygen atoms and more aromatic rings, which means that it can be easily converted to carbon materials without forming many micropores [39]. The composition of biomass has a great influence on the decomposition process. Chemical transformation of HTC biomass generally consists of three steps: (1) hydrolysis of biomass to (hydroxymethyl) furfural; (2) polymerization to form polyfurans; and (3) carbonization by further intermolecular dehydration. Cellulose hydrolysis is more likely to form glucose, while lignin can be decomposed into its main product, phenol. As for hemicellulose, the HTC process is more complex. For example, 504 reactions involving 114 pyrolytic species were found in the pyrolysis of hemicellulose. As HTC continues, the oxygen and hydrogen content (assessed by O/C and H/C ratios) will decrease due to demethanation, dehydration and decarboxylation [40].

In addition to HTC and pyrolysis, other methods such as microwave [41, 42], plasma [43, 44], laser [45–47] and flash joule heating [48] have been used to convert biomass into carbonaceous materials. Omoriyekomwan et al. [49] reviewed the microwave-assisted pyrolysis of biomass to produce carbon nanotubes and carbon nanofibers. Cellulose and polysaccharides are active biological components responsible for the generation of carbon nanofibers and carbon nanotubes from biomass by microwave irradiation. In contrast to HTC and pyrolysis, plasma provides not only high temperatures and pressures, but also an abundance of free radicals, ions and molecules from the biomass, which can rapidly decompose biomass into simpler graphene [50]. Shah et al. [43] used plasma after pyrolysis to clarify the importance of plasma for graphene growth. Despite the decomposition of biomass by breaking chemical bonds during pyrolysis at 750 °C, macromolecules formed from pyrolytic debris are not conducive to graphene growth. Once the plasma is turned on, carbon atoms, ions and free radicals are deposited on the copper substrate and the resulting hydrogen radicals also etch and clean the oxides from the copper substrate.

Lasers can also induce local high temperatures and pressures as a common laser cutting tool for converting biomass to graphene. During laser irradiation [46], hemicellulose and cellulose decompose more easily and produce more defects under laser irradiation. At the same time, aromatic lignin can form graphene to a lower degree. It should be noted that the laser-treated raw lignin formed amorphous carbon with high viscosity, indicating the importance of the crosslinked lignocellulosic structure. South Korea and others. Graphene embedded with metal nanocrystals was further synthesized by laser conversion of biomass [46]. However, Mahmood et al. [47] prepared porous graphene derived from lignin by laser writing. The lignin film was firstly prepared with water-soluble cork alkaline lignin and protected with adhesive tape to avoid deformation of the film at high temperature. During laser writing, gaseous molecules from lignin lead to the formation of porous structures. The photothermal process is charged by laser intensity/power. Layered porous carbon and graphene carbon can be obtained by laser irradiation with an optimal power range. With increasing laser power, ordered graphene structures with rich defect boundaries can be observed.

### Biomass-Based Material Structure and Applications

Biomass feedstock directly affects the performance and application of biomass char by influencing the composition and structure of biomass char, and to some extent plays a decisive role in the activity and reactivity of biomass char. Specifically, the catalytic performance and reactivity of biochar are mainly controlled by its surface-active sites (e.g., metal atoms, non-metal heteroatoms, surface-active functional groups, defective structures) [51]. These active sites are essentially composed of elements, which are reflected in the surface structure of biochar and influenced by the carbon structure and porosity. It is by influencing these components that different biomass feedstocks affect the performance of their corresponding biochar.

It is worth noting that the elemental composition of biochar from various sources differs due to the functional composition of the biomass itself, further resulting in differences in the types of biochar activity. One of the most prominent features of sludge-derived biochar is its higher ash content than plant and animal sources, which may be due to the complexity of sewage sludge and the diversity of its composition. Its ash content consists mainly of silica compounds and various heavy metal components (e.g., Ni, Cu, Cr, Cd and Pb) [52]. Several studies have also shown that N doping and metal (e.g., Fe and Al) loading are common in sludge-derived biochar. The reason for this phenomenon is the addition of flocculants (e.g., polyacrylamide, polymerized ferric sulfate, polymerized aluminum chloride) during the chemical conditioning of the sludge. As a result, sludge-derived biochar may often contain more significant reactivity imparted by transition metals. However, ash from sludge-derived biochar is often difficult to clean and often requires high-risk reagents (e.g., hydrofluoric acid and nitric acid) to etch or restructure the sludge-derived biochar to expose the active structure covered by the ash [53].

The biochar surface is the main site of interfacial reactions, including interfacial adsorption and catalysis. Surface properties mainly include specific surface area (SSA), surface functional groups (SFGs), and surface electrical properties, depending on production parameters (e.g., pyrolysis temperature and time) and biomass type [54]. The increase in SSA of biochar during carbonization is due to the growth of nanopores generated by the growth of high-density vortex layer microcrystals. The SSA of plant biomass char was higher than that of other charcoals, while the SSA of sludge biomass char was the lowest, probably due to the higher ash content covering the pores of sludge biomass char. It is noteworthy that the SSA of bone-derived biochar is higher than the first two. This may contribute to the excellent porosity configuration of biochar in bone constructed from natural pore templates and acid etching to further modify the pore structure.

In contrast, the SSA of manure-derived biochar was only 0.02–0.16 times higher than that of lignocellulosic biochar [55]. The surface-active functional groups of biochar are the most intuitive manifestation of its adsorption and catalytic properties. The surface-active components, however, are mainly composed of elements derived from biomass and vary with the abundance and species in different biomass sources. In other words, biomass can impart different surface activities to biochar by influencing the type and abundance of SFGs in the resulting biochar. For example, the –COO antisymmetric stretch of amino acids is often found in wood and crop waste biochar, and CO32– is usually found in waste and manure biochar and rarely in biochar of plant origin [56].

The carbon composition of biochar can be divided into dissolved organic carbon and polymeric carbon skeletons, with the latter predominating. Specifically, the carbon skeleton consists of relatively disordered *sp*^2^ and *sp*^3^ hybridized carbons that exhibit an amorphous structure. Based on their graphitization and arrangement, typical aromatic cluster models and rectangular polycyclic aromatic ring-like models are proposed to describe the carbon skeleton morphology [57]. In general, the preparation parameters that can induce carbon arrangement (e.g., pyrolysis temperature) usually have a greater effect on the carbon skeleton form than the biomass type.

Regardless of the biomass type, non-carbonized organic matter in the carbon skeleton structure (including amorphous lignin, crystalline cellulose and amorphous hemicellulose, dominated by *sp*^3^ hybridization) slowly transforms to amorphous aromatic carbon and then to conjugated aromatic carbon as the pyrolysis temperature increases [58]. In addition, dissolved organic carbon (DOC) is also an important component of biochar, and DOC affects the stability and performance of biochar-based materials in the environment. Further studies have shown that biomass type can influence the nature of DOC, which is of interest for the application of different types of biochar [59]. The pore structure is not only the basis for the expression of adsorption activity of biomass char, but also an important contact or attachment site for the catalytic process, which is strongly influenced by the original template structure, density, and ash composition of the precursor biomass feedstock.

The average pore size of biochar typically ranges from 2 to 50 nm and varies depending on the biomass type. For animal bones, nanoscale plate-like hydroxyapatite crystals are dispersed in discrete spaces of collagen fibers. Unlike fecal-derived biochar, organic matter in bones can act as carbon precursors and natural minerals can act as their own templates, playing a crucial role in the formation of mesopores and macropores [60]. Animal bones are layered composites with concentric sheets or plywood-like sheets that can be used to form the layered structure of biochar. In addition, animal shells such as shrimp shells also have properties similar to animal skeletons and can form clearly layered porous structures [61]. For biochar of plant origin, lignin-rich biomass (e.g., bamboo, coconut shells) tends to form biochar with larger pores, while biochar prepared from cellulose-rich biomass (e.g., rice husk) usually has smaller pore sizes [62].

Biochar produced from cellulose-rich peel mesocarp exhibited a lamellar morphology and higher porosity compared to lignin-rich peel exocarp. Therefore, it is reasonable to speculate that biochar produced from cellulose-rich biomass has a finer pore structure and higher pore density. In addition, the pore classification of biochar obtained from different biomass feedstocks differed somewhat. Plant-derived biochar (e.g., sawdust and straw) and sludge-derived biochar mainly contain micropores and mesopores, while animal-derived biochar (e.g., pig bone biochar and eggshell biochar) are dominated by mesoporous structures, some of which (e.g., shrimp shell) even have three pore structures (micropores, mesopores and macropores) [63].

Aside from the various raw material utilizations in natural biomass, artificial composition management and structural design at various scales also show remarkable value in material synthesis and application. The hydroxyl group on the surface of wood cellulose allows for increased mechanical strength. According to studies [64], after removing a considerable amount of lignin and hemicellulose with a combination of sodium hydroxide and sodium sulfite, the exposed hydroxyl groups of a big amount of cellulose and hemicellulose could form multiple hydrogen bonds with each other at high temperatures. The elimination of lignin and hemicellulose enhances not only the density of wood, but also its mechanical qualities due to the production of a significant number of hydrogen bonds. This technology produces extraordinarily strong wood (548.8 MPa, ten times stronger than natural wood), which means it could one day be utilized as a less expensive, greener alternative to steel or even titanium alloys. Similarly, increasing the number of hydroxyl groups exposed to the cellulose/hemicellulose chain would allow for more chemical changes. Li et al. [65] extracted lignin from natural wood and modified its surface charge to generate a nanofluid film made of wood nanofibers and high-density cellulose, the conductivity of which increased from 1 to 2 ms cm^−1^ following modification. Furthermore, the membrane's nanofiber channel diameter may be varied between 2 and 20 nm, allowing for high flexibility and 150° folding. As a result, wood-derived cellulose membrane has a promising future in the field of folding and high-performance nanofluid devices.

### Biomass-Based Material Optimization

**Fig. 4:**
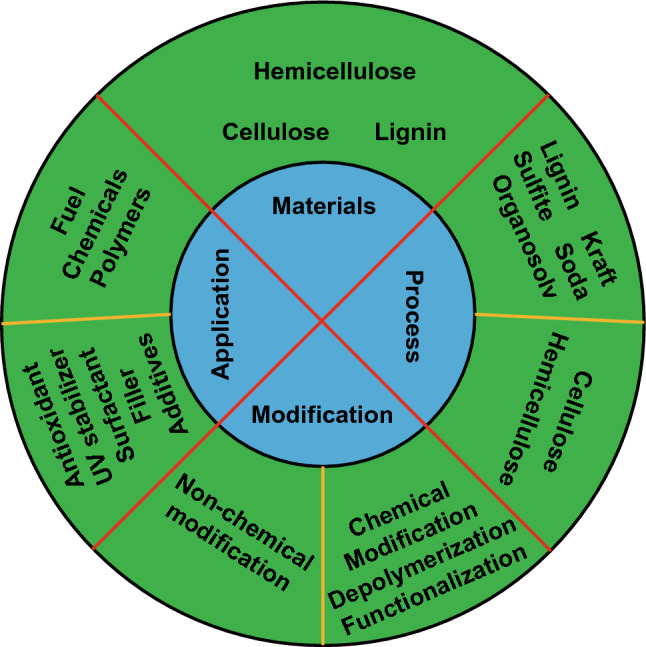
Schematic representation of the main processes for lignin extraction and possible chemical modifications performed in order to valorize lignin, depending on the applications [66]

The chemical structure of biomass can be easily modified to develop new materials, especially lignin rich in is phenolic and aliphatic hydroxyl groups. Lignin can be used with or without chemical modifications depending on the target application (Fig. 4) [66]. Without chemical conversion, lignin can be incorporated directly into polymer matrices to reduce production costs and improve performance. For example, unmodified lignin can be used as UV stabilizers, antioxidants, flame retardants and additives to promote plasticity and flowability of the final product [67–74]. Although there is potential for direct industrial application, unmodified lignin can only be incorporated in small amounts due to its weak mechanical properties. On the other hand, lignin can be chemically modified to be used as a starting material for polymer synthesis or for conversion into chemicals and fuels [75–78]. There are four different ways to chemically modify lignin: (1) lignin depolymerization or fragmentation, using lignin as a carbon source or cleaving it into small fragments containing aromatic rings; (2) modification of lignin by synthesis of new chemically active sites; (3) chemical modification of the hydroxyl groups presents in the lignin structure; and (4) production of graft copolymers. These chemical modifications are very dependent on the reactivity and structural characteristics of the functional groups of the lignin used [66].

**Fig. 5:**
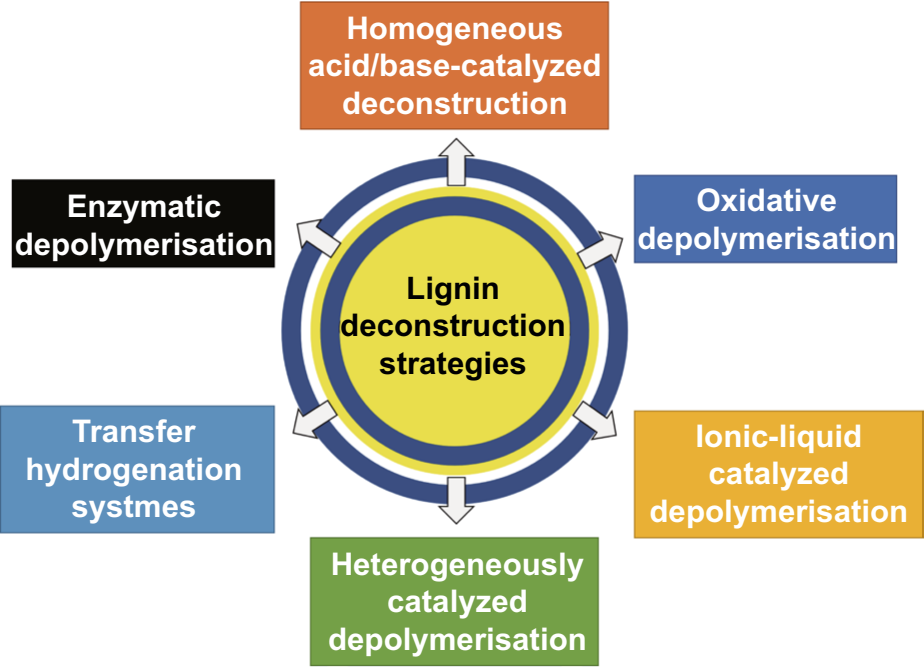
Schematic diagram of the deconstruction strategy of lignin [81]

Lignin depolymerization or fragmentation is a promising method to convert lignin feedstock and produce valuable lignin-based products. Thus, lignin molecules are converted into small compounds for further applications, including fuels and basic chemicals or oligomers [79]. Several thermochemical methods have been investigated for lignin depolymerization, such as pyrolysis, oxidation, hydrolysis and gasification (Fig. 5) [80].

**Fig. 6:**
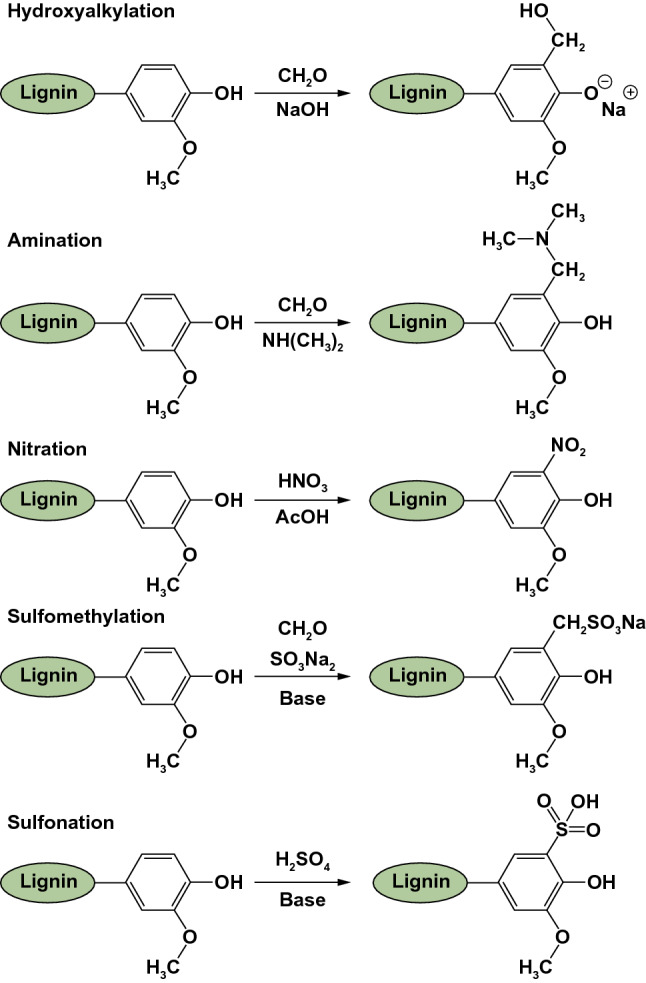
Overview of the chemical modifications of lignin: synthesis of new chemically active sites [82]

Lignin has several functional groups, including hydroxyl, methoxy, carbonyl and carboxyl groups. These functional groups can be modified for different applications, which will increase the value of modified lignin. These modifications include the synthesis of new macromolecular monomers that are more efficient and reactive by increasing the reactivity of the hydroxyl group or by changing the nature of the chemically active sites. As a result, the chemical reactivity of lignin is increased, the brittleness of lignin-derived polymers is reduced, the solubility of lignin in organic solvents is increased, and therefore lignin processing is improved. In this way, several chemical modifications have been used to introduce new chemical sites in the lignin structure (Fig. 6) [82], including hydroxyalkylation, amination, nitration, sulfomethylation, and sulfonation [82–85].

Lignin has phenolic and aliphatic hydroxyl groups present in its structure in the side chains. The phenolic hydroxyl group is the most reactive functional group and can influence the chemical reactivity of the newly formed material. Modification of the hydroxyl groups can lead to the formation of polyol derivatives of lignin. For this reason, several reactions to functionalize lignin with different functional groups have been reported and studied, these include alkylation, esterification, etherification, phenolization and carbamylation reactions [86–90]. In addition, lignin can be used to develop lignin graft copolymers in which polymer chains are attached to hydroxyl groups on the lignin structure, resulting in a star-shaped branched copolymer with a lignin core [91–94] (Tables 1 and 2).

Similarly, cellulose material modification can solve usage-related faults such as poor heat formability, excessive hydrophilicity, and poor mechanical properties [95–97]. The primary hydroxyl group on C-6 and the subsidiary hydroxyl groups on C-2 and C-3 of the glucose ring units of cellulose afford it an extensive range of modification options. It can be subdivided into etherification, crosslinking, esterification, grafting copolymerization, and so on, based on the introduction of various functional groups and their degrees of substitution at various positions of the glucose group ring unit. The modification of the hydroxyl group on its molecular chain by esterification with acid or acid derivatives is known as esterification modification [98] (anhydride, acyl chloride, etc.). The degree of cellulose substitution was controlled by varying the amount of acid or acid derivatives, catalyst concentration, reaction time, reaction temperature, and reaction medium, resulting in prime derivatives with varying properties. Etherification, like esterification, can improve the characteristics of cellulose or add new functions to cellulosic materials. Typically, epoxide and chloroalkyl groups are accelerated to create ether bonds by reacting with hydroxyl groups on the cellulose skeleton. Esterification is primarily utilized to improve the solubility, biodegradability, and application performance of cellulose. Because ether bonds are substantially more stable under basic conditions than ester bonds, cellulose ether derivatives offer a broader range of applications [99]. The active hydroxyl group at the end of the cellulose molecular chain is polymerized with the monomer under the action of the initiator [100], and new functional groups are introduced into the polysaccharide molecular chain through grafting polymerization to improve its characteristics [101]. The reaction of cellulose grafting copolymerization can be classified into three mechanisms: ring-opening polymerization, free radical polymerization, and ion polymerization. Cellulose materials that have been grafted and polymerized have improved biocompatibility, conductivity, and water resistance. Crosslinked cellulose is cellulose that has been changed by the connection of two or more cellulose molecules via a tiny molecule. The crosslinking agent has several active groups and can be linked to cellulose via chemical reactions such as etherification, esterification, and free radical polymerization. Crosslinking agents that are often utilized include epichlorohydrin [102], dicarboxylic acid [103], diisocyanate [104], and diacrylate [105]. To create interpenetrating polymer networks, cellulose can be crosslinked with other polymers such as PVA [106], chitosan [107], sodium alginate [108], and others. This is another method for incorporating the characteristics of other polymers into cellulosic products.

## Biochemical Conversion

Numerous studies have focused on BC of biomass and materials, with BC of biomass studies focusing on lignocellulosic biomass feedstocks. Lignocellulosic biomass is of high interest to produce biofuels and bioproducts via the process of enzymatic hydrolysis [109]. Besides, lignocellulosic biomass such as corn stover was scrutinized to produce bio-succinic acid via biological routes of pretreatment, fermentation, as well as downstream technologies for bio-succinic acid recovery [110]. Furthermore, lignocellulosic feedstocks like corn cobs are of high potential to produce bioethanol with a prominent yield of 0.49 g g^−1^ biomass via the processes of NaOH pretreatment, hydrolysis and fermentation [111]. Integrated biorefinery including pretreatment, enzymatic hydrolysis and fermentation was applied for conversion of agricultural residues, forest waste, energy crops into second-generation bioethanol as well as value-added products [112]. There are also studies center of attentionon is feedstock of microalgal biomass. BC of microalgal biomass studied for useful and cost-effective biofuels [113]. Microalgae have been also investigated to produce bio-stimulants and biofertilizers via anaerobic digestion [114]. For example, mixed microalgae have been shown to be a substrate for a 17 mL g^−1^ COD biomethane production through an anaerobic digestion process [115]. In the case of biological hydrogen production, mixed microalgae with a biomass concentration of 2.57 g L^−1^ can recover 13 mL g^−1^ biohydrogen at the laboratory scale level [116]. In addition, some researchers have implemented pilot-scale reactors to increase output and productivity. For example, biomethane production of 0.27 L CH_4_ g^−1^ VS was achieved with mixed microalgal biomass in a pilot-scale high-rate cyclotron algal pool [117]. Heterogeneous catalytic biochemical processes have been reported for the efficient production of value-added chemicals and biofuels from both edible and non-edible biomass [115]. Biological lignin valorization was applied to produce polyhydroxyalkanoates having a selling price of 6.18 $ Kg^−1^ [116]. Microbial fermentation and alkaline hydrolysis of sugarcane bagasse were studied to produce high concentration of l-tyrosine from a newly p-coumaric acid biotransformation route [117]. In drying biomass there has been a large amount of energy utilized, so if biomass has moisture content less than 50% then for generation of energy direct combustion method recommended. If it is present more than 50% in wet biomass than BC is most valuable and promising method to convert biomass into biofuel. In general, BC of biomass is highly vital for the supply of biofuels and biomaterials.

## Electrochemical Conversion

EC is of high reputation as a cleaner technology to produce sustainable energy and materials. Electrochemical process uses electrons as reactants, habitually works at low temperature (in most cases below 100 °C) and ambient pressure, which is considered as low-cost, environmentally benign, and “green” [120]. Electrocatalytic synthesis of high value-added chemicals from bioresource is a research hotspot [121]. EC of biomass-derived intermediates (e.g., lignin, polyols, carboxylic acids, furans, and amino acids) has been emphasized to produce fuel, chemicals, as well as materials [122]. In a recent study, EC of biomass-derived compounds was investigated for synthesis of 4-ethylnonane, which contributed to a high product yield of 94% with an apparent coulomb efficiency of 4700% [123]. With this, biomass-derived feedstock levulinic acid was investigated to produce 4-hydroxyvaleric acid via outer sphere electron transfer route [124]. The outcomes showed that 4-hydroxyvaleric acid reached a production rate of more than 40 g (L h) ^−1^. Besides, the selectivity was higher than 99.9%, and conversion as well as faradaic efficiency were both more than 80%. Non-Kolbe electrolysis of bio-derivable hydroxy acid was studied to produce C9 oxygenate mixture with high-quality fuel properties with a density of 834 kg m^−3^ at 15 ℃ [125]. Furthermore, biomass-derived compounds like aldehydes, aryl ethers and phenolic compounds were studied for value-added hydrocarbons by electrocatalytic processes with desired selectivity and conversion efficiency [126]. Electrocatalysis of biomass-derived 5-hydroxymethylfurfural (HMF) was carried out to produce 2,5-diformylfuran (DFF) with a separation rate of 78% and a selectivity of 100% [127].

While co-electrocatalytic conversion process has been settled in recent years, furan coupling electrolysis of biomass-derived furfural was of interest for hydrofuran production with a yield of 94% and a faradaic efficiency of 93% in batch electrolytic cell [128]. Additionally, electrochemical decarboxylation and cross-coupling of biomass-based carboxylic acids were examined for sustainable fuel production in presence of (Ru_*x*_Ti_1−*x*_)O_2_ catalyst [129]. The findings showed that the conversion of α-methylsuccinic acid reached 89–96% while the maximum yield of methyl 2,5-dimethylhexaneoate (MDH) and 2,5-dimethylhexaneoate (DH) achieved 37 and 21%, respectively. The unconfined carbon dioxide can be used in molten carbonate fuel cells and has great potential for further application. Simultaneous electrocatalytic oxidation (ECO) and electrocatalytic hydrogenation (ECH) of 5-hydroxymethylfurfural (HMF) were coupled to generate 2,5-furandicarboxylic acid (FDCA) with a high selectivity of more than 96%, and a faradaic efficiency of higher 84% [130]. More interestingly, nanostructured NiFe oxide (NiFeOx) and nitride (NiFeNx) catalysts were used to assemble an electrolytic cell to electrolyze glucose [131]. The results showed a glucaric acid yield of 83% and a faradaic efficiency of 87%. Besides, electrochemical reduction of glucose produced gluconic acid at a cost of 54% lower than current chemical methods.

EC of lignin was handled for green chemicals and materials via hybrid of electro-oxidation, electroreduction, electrochemical upgrading, and fractionation [132]. Biomass-derived products like glycerol, 5-hydroxymethylfurfural, levulinic acid, and muconic acid were efficiently converted to high-value chemicals of glyceraldehyde, glyceric acid, formic acid, glycolic acid via EC [133]. Waste biomass of bread residue, cypress sawdust, and rice chaff were electrolyzed with 85% phosphoric acid solvent at 150 °C [134]. The current efficiency of hydrogen produced by the cathode was about 100%. In all the tested fuels, hydrogen production was 0.1–0.2 mg mg^−1^ feedstock. It is evident that glycerol, furfural, 5-hydroxymethylfurfural, levulinic acid and other biomass-derived substances are mostly used as starting reactants to synthesize fuel. It is important to emphasize on electrocatalytic synthesis of high value-added products from HALUB initially for economic availability. Focusing on HALUB, many investigations can be settled in the field for efficient and sustainable production of fuels, chemicals, and new materials.

### Electrochemical Valorization of Lignin

**Fig. 7:**
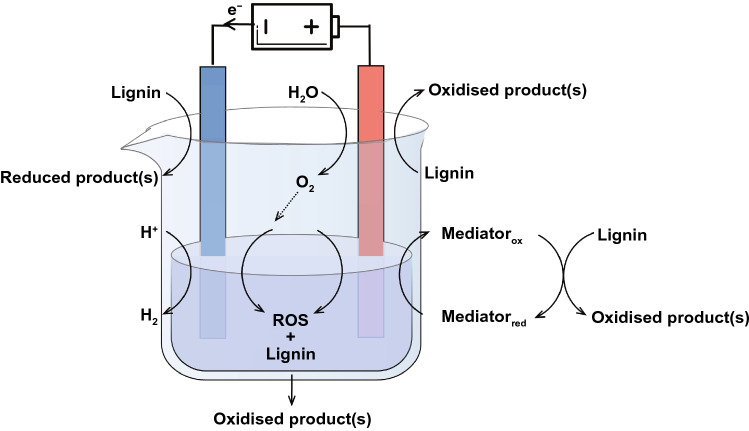
Electrochemical lignin valorization. Electro-oxidation at the anode via direct, mediated methods and via the generation of reactive oxygen species (ROS) from reduction of O_2_. Electroreduction via direct method. Water splitting competes with lignin electrochemical conversion at both anode (OER) and cathode (HER)

Various research activities, such as pyrolysis, chemical and enzymatic catalysis, have been conducted to degrade lignin into small molecules that have potential to be used as chemical feedstocks or fuels. Beyond research efforts, electrochemical depolymerization and upgradation offers an alternative for lignin valorization [138]. In light with the fact that renewable electricity from sources such as solar and wind energy becomes more abundant and cheaper, the electrochemical process has potential to further incorporate renewable energy into biomass utilization and associated industries [138]. Electrochemical lignin conversion can take place at both anode (oxidation reaction) and cathode (reduction reaction), although more anodic studies have been reported so far [122]. In a typical electrochemical setup, an anode and cathode are employed to abstract and inject electrons, respectively, and a solvent with supporting electrolyte is used to dissolve lignin and conduct ions (Fig. 7).

In order for the lignin to be in close contact with either anode or cathode, the electrolyte should dissolve lignin well. However, the complicated structure of lignin renders its poor solubility in common organic solvents [138]. Alkaline solutions, such as NaOH and KOH, have been widely rummage-sale as the electrolyte in many studies of lignin electro-conversion owing to increased lignin solubility and high ion conductivity [122]. The main challenge is the competing water splitting reaction, i.e., oxygen evolution reaction (OER) at anode and hydrogen evolution reaction (HER) at cathode, which competes with lignin electroreduction and electro-oxidation, respectively, resulting in lower electro-conversion efficiency of lignin. Ionic liquids (ILs) have been used as alternative electrolytes because of their high lignin solubility and, more importantly, wide potential window to avoid water splitting reaction [139, 140] A range of ILs have been studied in electrochemical lignin depolymerization, for example, 1‐ethyl‐3‐methylimidazolium trifluoromethanesulfonate, 1-butyl-3-methylimidazolium tetrafluoroborate, and triethylammonium methanesulfonate [141, 142]. A barrier that averts ILs from commercial applications is the high cost of ILs [143].

At the anode side, lignin can be electro-oxidized via three primary ways, i.e., direct, mediated, and electrochemical or chemical combined strategies. Inclusively, the electro-oxidation involves lignin functionalization, depolymerization, and other side reactions. In the direct electro-oxidation, a heterogeneous catalyst is needed to adsorb and thereafter active the lignin via the break of C–O or C–C bonds, and therefore is the key. The electronic structures, morphologies, and surface areas of the electrocatalysts have marked effect on the activity, selectivity, and deactivation of lignin electro-oxidation. Various transition metals (e.g., Fe, Co, Ni), metal alloys, and metal oxides (e.g., PbO_2_, IrO_2_, and SnO_2_) have been studied [144, 145]. Mediated electro-oxidation of lignin uses mediators, which are usually homogeneous, to mediate the activation of lignin. This is essentially an out-sphere electrochemical reaction, involving an electrochemical oxidation of the mediator and the ensuing chemical oxidation of lignin by the oxidized mediator [146, 147]. For this reaction, the reaction regions can be extended from the two-dimensional interfaces in the direct electro-oxidation case to three-dimensional electrolyte phases. Polyoxometalates,* N*‐hydroxyphthalimide (NHPI), 2,2,6,6‐tetramethylpiperidine‐*N*‐oxyl (TEMPO), ferric, and halide have been widely studied as mediators [148]. In the electrochemical and chemical combined reaction, lignin in the electrolyte is both directly electro-oxidized at the anode and chemically oxidized by electro-generated reactive oxygen species (ROS, e.g., H_2_O_2_, O_2_^− ^⋅ and ⋅OH) formed at anode or cathode [149].

While oxidation usually surges the oxygen content, reduction of lignin increases hydrogen and carbon content in the final products, which is critical for the production of lignin-derived oil due to higher oxygen content reduces combustion value of the oil [150]. Electro-reduction, or electrochemical hydrogenation in some reports, uses protons and electrons to selectively add hydrogen or remove oxygen from lignin. This process involves the reduction of protons to form adsorbed H* (* denotes adsorbed species), which can either dimerize to desorb as gaseous hydrogen, i.e., HER or react with lignin to cleave C–O single bonds and add hydrogen to aromatic rings [151]. Thus, the HER is the major competing reaction with electro-reduction of lignin, decreasing overall energy efficiency of the process. The yield and selectivity of products from lignin electroreduction can be tuned by varying the nature of the cathode catalyst, current density, electrolyte, temperature, or mediator [132].

Electrochemical approach can be combined with other progressions for lignin valorization. For example, it can integrate with micro-organism to form microbial fuel cells (MFCs), microbial electrolysis cells, and electro‐microbial systems [152]. A solar thermal electrochemical process that embedded the use of solar energy has also been demonstrated to produce biofuels and hydrogen from lignin [153, 154]. Several challenges must be addressed for the electrochemical process to be commercially viable. Either electro-oxidation or -reduction suffers from competing reactions of water splitting and product selectivity from electrochemical lignin conversion is low, resulting in low energy efficiency and adding cost for additional product separation. Developing more effective and selective catalysts is critical, which involved more understanding of catalytic mechanism to guide design of new materials [155]. Further, the choice of electrolyte is limited to mainly alkaline solutions or ILs. Low-cost yet green solvents must be further explored.

### Microbial Fuel Cells

Microbial fuel cell (MFC) is a technology that renovates the chemical energy in biomass to electric energy relying on the catalytic action of microorganisms. MFC can use bioresources like microalgae for the generation of electricity and the treatment of wastewater or seawater. It has the dual functions of waste treatment and generation of electric energy. The information on MFC with HALUB biomass is tabulated in Table 3. Various biomass feedstocks have been investigated for use in MFCs, such as *Microcystis aeruginosa*, sugarcane biomass, cyanobacterial biomass, azolla pinnata biomass, and *S. japonica* substrate. The harmful algal biomass was applied as a substitute substrate for the anodic microorganisms in MFC for biological power generation [156]. The maximum power density, current density, and coulombic efficiency were 83 mW m^−2^, 672 mA m^−2^ and 7.6%, respectively. Sugarcane biomass was converted using acetate and *p*-coumaric acid as substrate in a MFC, achieving a potential of 0.34 V, and power density of 398 mW m^−2^ [157].

**Table 1:** Biochemical conversion to energy and materials

Feedstock	Approach	Performance	References
Lignocellulosic biomass	Enzymatic hydrolysis	Biofuels and bioproducts	[109]
Lignocellulosic biomass	Biological routes	Bio-succinic acid production, selling price 1.7–2.0 $ Kg^−1^	[110]
Lignocellulosic feedstocks	Fermentation	Bioethanol yield 0.49 g g^−1^·biomass	[111]
Agricultural residues, forest materials and energy crops	Pretreatment, enzymatic hydrolysis and fermentation	Biofuel and value-added products	[118]
Microalgal biomass	Nanoparticle-assisted biochemical conversion	Attained the highest amount of COD of 14,760 and 14,745 mg L^−1^ using Fe and Ni NPs respectively	[119]
Microalgae	Anaerobic digestion	Bio-stimulants and biofertilizers	[114]
Edible and non-edible biomass	Heterogeneous catalytic biochemical processes	Value-added chemicals and biofuels	[115]
Lignin	Pretreatment, solubilization and enzymatic hydrolysis	Polyhydroxyalkanoates $6.18 kg^−1^	[116]
Sugarcane bagasse	Microbial fermentation and alkaline hydrolysis	l-tyrosine 49% and *p*-coumaric acid 44 mg	[117]

**Table 2:** Electrochemical conversion biomass to energy and materials

Feedstock	Approach	Performance/Products	References
Biomass-derived intermediates	Low-temperature, electrochemical reduction, and oxidation	Fuels, chemicals, and materials	[135]
Coupling biomass-derived C_5_/C_6_ compounds	Electrochemically induced coupling reaction of 2-MF and 3-hexene-2,5-dione (HEO)	Branched alkane 4-ethylnonane 91% yield, apparent coulomb efficiency 4700%	[123]
Biomass-derived feedstock levulinic acid	Three-electrode H-type cell, outer sphere electron transfer route	4-hydroxyvaleric acid production rate > 40 g (L h) ^−1^, > 99.9% selectivity, conversion and faradaic efficiency > 80%	[124]
Bio-derivable hydroxy acid	Non-Kolbe electrolysis of a biogenic acid containing a 3-hydroxy backbone	Density of C_9_ oxygenate mixture complies 834 kg m^−3^ at 15 ℃, flash point 58.5 ℃ (in line with the current statutory diesel specification EN590)	[125]
Biomass-derived compounds	Electrocatalytic processes, multiple electrolytes, and electrode materials, coupling water electrolysis	Value-added hydrocarbons and hydrogen	[126]
Biomass-derived 5-Hydroxymethylfurfural (HMF)	Electrocatalysis	Separation rate of HMF to DFF 78%, selectivity of 100%	[127]
Biomass-derived furfural	Furan coupling electrolysis in a basic electrolyte (0.1 M KOH)	Yield of hydrofuran 94%, Faraday efficiency 93% in batch electrolytic cell	[128]
Biomass-based carboxylic acids	Electrochemical decarboxylation and cross-coupling	Conversion of α-methyl succinic acid 89–96%, maximum yield of MDH 37%, maximum yield of DH 21%	[129]
5-Hydroxymethylfurfural (HMF)	Electrocatalytic oxidation (ECO) and electrocatalytic hydrogenation (ECH) of HMF	HMF conversion ≥ 98%, FDCA selectivity ≥ 96%, faradaic efficiency ≥ 84%	[130]
Glucose	Electrolyze glucose	Faradaic efficiency 87%, glucaric acid yield 83%, electrochemical reduction of glucose produces gluconic acid at a cost 54% lower than current chemical methods	[131]
Lignin	Electro-oxidation, electroreduction, electrochemical upgrading, and fractionation	Metal/metal alloy, anode materials, dimensionally stable anodes (DSAs; metal oxide/ mixed metal oxide)	[132]
Biomass-derived products	Electrochemical conversion	Glycerol, dihydroxyacetone, 2,5-bis (hydroxymethyl)furan, 2-hydroxymethyal-5-methyal furan	[133]
Waste biomass (bread residue, cypress sawdust, and rice chaff)	Electrolysis with 85% phosphoric acid solvent at 150℃	Current efficiency of hydrogen produced by the cathode about 100%, hydrogen production 0.1–0.2 mg mg^−1^ feedstock	[134]
Glycerol	Electrocatalytic oxidation of glycerol by CuCo_2_O_4_ spinel oxide nanostructure catalysts	Selectivity for formic acid production 80.6%, overall Faradaic efficiency toward all value-added products 89.1%, glycerol conversion 79.7%	[136]
Lignosulfonate (LS)	Electrocatalytic oxidation	Capacitance properties of novel multifunctional hybrid materials 732.5 F g^−1^@0.5 A g^−1^, superior electrocatalytic activity for OER 264 mV@10 mA cm^−2^, HER 291 mV@10 mA cm^−2^	[137]

**Table 3:** Microbial fuel cells meet with HALUB

Feedstock	Approach	Performance	References
Microcystis aeruginosa	Harmful algal biomass as a substitute substrate for the anodic microorganisms in MFC algae for biological power generation	Maximum power density 83 mW m^−2^, current density 672 mA m^−2^, coulombic efficiency (7.6%)	[156]
Sugarcane biomass	Using acetate and p-coumaric acid as substrate in MFC	The potential 0.34 V, power density 398 mW m^−2^	[157]
Cyanobacterial biomass	A double chamber MFC with *Anabaena vaginicola* cyanobacterial biomass as the anodic substrate	Highest current density 366 mA m^−3^, maximum power density 144 mW m^−3^, chemical oxygen demand removal efficiency 65%, coulombic efficiency 5.7%	[158]
Azolla pinnata biomass	Using azolla biomass as the substrate and defatted azolla biochar as the electrode	Voltage 382 mV, COD decreased by 65.6%, simultaneous production of biological hydrogen and bioelectricity	[159]
*Exoelectrogenic yeast strain*	Using xylose as the substrate in an MFC	Power output 67 mW m^−2^, hydrogen gas 23 L m^−3^	[160]
*S. japonica*	A hybrid process of dark fermentation and MFC	Energy recovery of 17.3%, H_2_ yield 110 mL g^−1^·VS, maximum power density 1.82 W m^−2^	[161]
Sludge fermentation liquid (SFL) and fruit waste extracts (FWEs)	Microbial synergy with sludge fermentation fluid and fruit waste extract, bioelectricity produced through MFC	Peak output voltage of the hybrid MFC 0.75 V, the bioelectric energy conversion efficiency 1.391 kWh Kg^−1^·COD	[162]
Sludge fermentation liquid (SFL)	Microbial synergy with sludge fermentation fluid, and bioelectricity produced through MFC	Peak output voltage of the hybrid MFC 0.65 V, the bioelectric energy conversion efficiency 1.061 kWh Kg^−1^·COD	[162]
Fruit waste	Using zinc and copper electrodes and *H. undatus* as the substrate in an MFC	Maximum power density 0.072 W cm^−2^, current density 0.051 A cm^−2^	[163]
Fruit waste	Using zinc and copper electrodes and *R. ulmifolius* as the substrate in an MFC	Maximum power density 0.0668 W cm^−2^, current density 0.025 A cm^−2^	[163]

There was a study concentrating on a double chamber MFC with *Anabaena vaginicola* cyanobacterial biomass as the anodic substrate [158]. The results showed that the highest current density, maximum power density, chemical oxygen demand removal efficiency and coulombic efficiency reached 366 mA m^−3^, 144 mW m^−3^, 65, and 5.7%, respectively. Azolla pinnata biomass was inspected as substrate while defatted azolla biochar was applied as electrode. Biohydrogen and bioelectricity could be simultaneously generated in this system [159]. There were 67 mW m^−2^ power output and 23 L m^−3^ hydrogen produced from a new exoelectrogenic yeast strain that can generate electricity in MFC using xylose as the substrate [160]. An excellent energy recovery of 17.3% was obtained from *S. japonica* with H_2_ yield of 110 mL g^−1^ VS and maximum power density of 1.82 W m^−2^ in a hybrid process of dark fermentation and MFC [161].

In recent years, MFC was not only used in biomass conversion as declared above but also used in waste treatment. Microbial synergy with sludge fermentation fluid and fruit waste extract generated bioelectricity through MFC [162]. The peak output voltage of the hybrid MFC reached 0.75 V, while the bioelectric energy conversion efficiency achieved 1.39 kWh Kg^−1^ COD. Another study reported that MFCs were manufactured using zinc and copper electrodes and *H. undatus* as the substrate in the processing of fruit waste, showing the maximum power density of 0.072 W cm^−2^ and current density of 0.051 A cm^−2^ [163]. Although HALUB for MFC has been studied in open literature, some of the reported methods have limitations, such as low power density, coulombic efficiency, bioelectric energy, and conversion efficiency, particular for the feedstocks of biowaste. Future research could concentrate on the exploration of milder waste pretreatment unit that are compatible with MFC to enhance the performance.

### Fuel Cell System

Using fuel cells to treat waste biomass and generate electricity is a novel way to achieve the goal of waste treatment and energy recovery. The process requires converting the biomass to gas such as hydrogen or carbon monoxide before it can be utilized, so the cells are essentially unable to process biomass directly. There have been some studies absorbed on the fuel cell systems meeting with HALUB (Table 4) [164]. A study reported that the highest output power densities (*P*_max_) reached 12.3 and 41.8 mW cm^−2^ with open-circuit voltages of 560 and 1560 mV, respectively, through a liquid flow fuel cell under acidic and alkali conditions in processing of lignocellulosic biomass [165]. Another study found that power density and energy density achieved 440 mW cm^−2^, and 0.83 Wh g^−1^, respectively, from solid oxide fuel cell in processing of lignin, which performance was better than plastic wastes like polyethylene terephthalate [166]. A hybrid power system achieved a net output power of 196.2 kW, 1510 ton of CO_2_/year reduction and 21,901 $/year of environmental benefit, where the woody biomass gasification technology was integrated with the molten carbonate fuel cell, an externally fired gas turbine, and a supercritical carbon dioxide cycle [167]. The electrical power, heating power and cooling power reached 1000, 250, and 99 kW, respectively, in a novel rice straw based SOFC-Engine system for cooling, heating and power production [168].

**Table 4:** Fuel cell system meet with HALUB

Feedstock	Approach	Performance	References
Lignocellulosic biomass	Liquid flow fuel cell (LFFC)	Highest output power densities (P_max_) of the LFFC under acidic conditions 12.3 mW cm^−2^, P_max_ of the LFFC underand alkali conditions 41.8 mW cm^−2^	[165]
Lignin	Solid oxide fuel cell	Power density 440 mW cm^−2^, energy density 0.83 Wh g^−1^	[166]
Woody biomass	Hybrid power system where the biomass gasification technology is integrated with the molten carbonate fuel cell, an externally fired gas turbine, and a supercritical carbon dioxide cycle	Network output 196.2 kW, cost of electricity 0.1168 $ kWh^−1^	[167]
Rice straw	A novel biomass-based SOFC-Engine system for cooling, heating, and power production	Electrical power 1000 kW, heating power 250 kW, cooling power 99 kW	[168]
Rice husk ash (RHA)	White cement brick fuel cell (WC-BFC)	Maximum power density at Gr/Gr electrode 0.2 mW cm^−2^	[169]
Weed waste	Solid oxide fuel cell (SOFC)	Maximum power density 410 mW/cm^2^, maximum energy density of 0.42 W g^−1^	[170]
Palm mesocarp fiber (PMF)	Direct carbon fuel cell (DCFC)	Peak power density output 11.8 mW cm^−2^	[171]
Sugarcane bagasse	Liquid flow fuel cell (LFFC)	Power density 49.1 mW cm^−2^, co-generation of 101.4 Wh electricity	[172]
Sugarcane press-mud	Low-temperature proton exchange membrane fuel cell (LTPEMFC)	Energy efficiency 56%, renewability factor 1.24, carbon footprint 1.21 kg CO_2_ kW h^−1^	[173]

Biomass-based solid waste has recently been sightseen as a carbon neutral, sustainable and inexpensive feedstock in fuel cell system. White cement brick fuel cell was pragmatic to convert rice husk ash and achieve a maximum power density of 0.2 mW cm^−2^ at Gr/Gr electrode [169]. Another study found that the maximum power density and energy density reached 0.41 W cm^−2^, and 0.42 W g^−1^, respectively, via solid oxide fuel cell (SOFC) with the feedstock of weed waste [170]. Peak power density output achieved 11.8 mW cm^−2^ from direct carbon fuel cell in processing of palm mesocarp fiber [171]. In another study, liquid flow fuel cell was developed to convert sugarcane bagasse and achieve a power density of 49.1 mW cm^−2^, and co-generation of 101.4 Wh electricity [172]. Besides, low-temperature proton exchange membrane fuel cell was developed to process sugarcane press-mud and reached an energy efficiency of 56%, a renewability factor of 1.24 and a carbon footprint of 1.21 kg CO_2_ kW h^−1^ [173]. In brief, fuel cell would play an important role in processing of HALUB bioresource for efficient and renewable energy generation.

## Thermochemical Conversion

TC biomass to energy and materials is imperative for the carbon neutrality and environmental sustainability. Currently widely reported TC technologies include torrefaction, pyrolysis, hydrothermal carbonization, gasification, and liquefaction. This section is proposed to focus on the latest research progress. The information on TC biomass to energy and materials is summarized in Table 5.

**Table 5:** Thermochemical conversion biomass to energy and materials

Feedstock	Approach	Performance	References
Mixed agricultural and dairy waste	Co-pyrolysis a nutrient rich cow manure hydrochar with raw agricultural residues	Maximum specific surface area 48.8 ± 0.23 m^2^ g^−1^, pyrolysis bio-oils enrich in alkanes and alkenes with fewer oxygenated compounds	[177]
Livestock feces and biomass wastes	Co-pyrolysis of livestock feces (PM: pig manure, CM: chicken manure) and biomass wastes (WC: wood chips, BS: bamboo sawdust, RH: rice husk, and CH: chaff) at 600 °C	Biochar with lower pH value and electrical conductivity value by RH and CH treatment, biochar with better fuel characteristics and thermal stability by WC and BS, biochar with high calorific value and low aromatic H/C ratio by PM	[178]
Algal bloom microalgae	Integrated dark fermentation-hydrothermal liquefaction (DF-HTL)	In comparison to the control, the biocrude oil yield by 9.8%, carbon content (mol) by 29.7, energy content (MJ) by 40.0, energy conversion ratios by 61.0%	[179]
Cow manure	Hydrochar-impregnation (HAC), impregnated biomass pyrolysis (PAC) and one-pot HTC	Maximum adsorption capacity of phenol HOC 102.72 mg g^−1^, HAC 42.12 mg g^−1^, PAC 125.70 mg g^−1^; HOC and PAC with higher amounts of surface functional groups, HAC with more C-O bond	[180]
Cherry pomace	Hydrothermal treatment followed by CO_2_ gasification	Gasification activation energies of biochar 668–732 kJ mol^−1^	[181]
Seaweed	Alkaline thermal treatment	High-purity 69.69 mmol-H_2_/(dry-ash-free) g-brown seaweed, conversion 71%	[182]
Pinewood shaves	Thermochemical liquefaction (solvents and acidic catalysts at mild temperatures and atmospheric pressure)	Conversion rate of cyclic carbonate synthesized from glycerol 98.7%, conversion rate of propylene glycol 79.4%, bio-oil with excellent thermal properties	[183]
Agricultural wastes (i.e., rice straw and corncob)	Thermochemically treated with 1.0 M HCl as the acid catalyst and *Alcaligenes faecalis*	Conversion rate 48.2%, maximum 11.3 mM 4-hydroxyvaleric acid through chemoenzymatic valorization	[184]

Co-conversion of different feedstocks is currently popular in thermochemical process. The all-out fatty acid methyl esters (FAMEs) yield of 95% achieved under the conditions of 80 °C, 20 min, dimethyl carbonate ratio of 6:1, catalyst ratio of 3% and methanol addition of 3% via chemical co-conversion of algal oil, waste cooking oil and dimethyl carbonate [174]. The maximum methane of 394.6 mL g^−1^ VS was obtained via co-digestion of algal biomass and food waste [175]. An experimental and numerical study was carried out on co-conversion of algal bloom and water hyacinth via Ni_2_P-loaded zeolite catalytic pyrolysis [176]. The results showed that hydrocarbon production of 70.23%, bio-oil HHV of 35.72 MJ Kg^−1^ and total benefit of 749 $/ton biomass were achieved under the conditions of algal bloom to biomass ratio of 0.40, catalyst to biomass ratio of 0.95, steam to biomass ratio of 3.75, 500 °C, and CO_2_ flow rate of 40 mL min^−1^. It is evident that the co-conversion process is hence of important interest to produce sustainable fuels and materials.

Dairy manure and agricultural waste were explored to verbalize nutrient-enriched products via hydrothermal carbonization and pyrolysis. The maximum specific surface area of produced biochar reached 48.8 m^2^ g^−1^, while the produced bio-oil was enriched in alkanes and alkenes with fewer oxygenated compounds [177]. Co-pyrolysis of livestock feces and biomass wastes with different blending ratios was implemented at 600 °C [178]. The results showed that biochar with lower pH value and electrical conductivity value can be obtained by rice husk and chaff treatment. Wood chips and bamboo sawdust had positive effects on improving the fuel characteristics and thermal stability of fertilizer biochar. Pig manure-based biochar had high calorific value and low aromatic* H*/*C* ratio. Furthermore, the formed carbide can be widely used as adsorbent or catalyst in the removal and conversion of metal and organic pollutants owing to its large specific surface area and porosity.

An integration of different conversion tactics is explored in HALUB management. The biocrude oil yield, carbon content, energy content and energy conversion ratio reached 9.8, 29.7, 40.0, and 61.0%, respectively, in comparison to the control via integrated dark fermentation-hydrothermal liquefaction in processing of algal bloom [179]. Carbonaceous materials resulting from hydrothermal carbonization and pyrolysis were characterized with higher amounts of surface functional groups, whereas carbonaceous materials derived from hydrochar-impregnation were detected with more C–O bond. The maximum adsorption capacity of phenol was 125.70 mg g^−1^ from the process of pyrolysis, while the value was 102.72 mg g^−1^ from hydrothermal carbonization [180]. Hydrothermal treatment and CO_2_ gasification of cherry pomace were conducted for the synthesis of carbonaceous materials. The highest yield and activation energy of the solid products reached 57% and 732 kJ mol^−1^, respectively [181].

The accumulation of external sources like solvent, catalyst or enzyme is beneficial to the thermochemical conversion process. Alkaline thermal treatment of seaweed was carried out to produce high-purity H_2_ [182]. The fallouts showed that H_2_ yield reached 69.69 mmol g^−1^·biomass with a conversion rate of 71%. Solvent-assistant thermal treatment is a promising approach to produce clean energy like hydrogen. Besides, thermochemical liquefaction of pinewood shaves was studied with solvents and acidic catalysts at mild temperatures and atmospheric pressure [183]. The conversion rate achieved 98.7% in presence of glycerol carbonate as a reaction solvent. The HHV of the obtained bio-oil was 27.88 MJ Kg^−1^, which was much higher than 19.35 MJ Kg^−1^ in absence of solvent. Agricultural wastes were thermochemically treated with 1.0 M HCl as the acid catalyst [184]. Through chemoenzymatic valorization, maximum 11.3 mM 4-hydroxyvaleric acid was obtained with a conversion rate of 48.2% in presence of *Alcaligenes faecalis*.

It is manifest that several biowastes were explored as the raw feedstocks in TC. Co-conversion of different feedstocks could be beneficial to generate high-value products partially owing to their variable physical and chemical characteristics. Additionally, a combination of varied processes like hydrothermal carbonization and pyrolysis, hydrothermal treatment and CO_2_ gasification, and fermentation and hydrothermal liquefaction, could highly enhance the energy conversion efficiency and product quality. It is of high interest for the application of pyrolysis, gasification, liquefaction, and carbonization in solid biowaste conversion and management for a green future.

### Hydrothermal Carbonization

Hydrothermal carbonization (HTC) is a promising know-how for converting high moisture feedstock to a safe low-emission hydrochar. HTC generally refers to wet torrefaction and can produce hydrochars with higher HHVs under lower temperature in short reaction time compared to that of dry torrefaction [185]. Different feedstock and approaches in HTC are listed in Table 6. HTC of sea lettuce produced 24.43% of hydrochar and the HHV reached 20.2 MJ Kg^−1^ under 220 °C for 2 h [186]. Two types of carbonaceous materials including oriented carbon microspheres (OCMSs) powder and 3D porous carbon (3DPC) block can be produced in one step by hydrothermal treatment of basswood block at 700 °C [187]. Sugarcane bagasse was converted at 200 °C for 18–20 h in a muffle furnace for HTC [188]. The maximum surface area of activated biochar was 1099 m^2^ g^−1^, and the surface has a mesoporous structure, which was rich in hydrophobic groups inside and hydrophilic groups outside. The adsorption affinity of active biochar for sulfamethoxazole (400 mg g^−1^) was higher than other materials.

**Table 6:** Different feedstock and approaches in hydrothermal carbonization

Feedstock	Approach	Product characteristics	References
Sea lettuce	150, 180, 200, and 220 °C for the residence time of 0.5, 1 and 2 h	Hydrochar 9.51–24.43% yield, the high heating value 13.4–20.2 MJ/Kg	[186]
Basswood	One step by hydrothermal treatment of basswood block at 700 °C	The as-assembled OCMS/3DPC potassium ion hybrid capacitor energy of 140.7 Wh Kg^−1^ at 643.8 W Kg^−1^ even over 8,500 cycles	[187]
Sugarcane bagasse	Heated at 200 °C for 18–20 h in a muffle furnace for HTC	Maximum surface area of active biochar 1099 m^2^ g^−1^ with mesoporous structure, adsorption affinity of active biochar for sulfamethoxazole 400 mg g^−1^	[188]
Simulated food waste	180 to 220 °C, 15 and 30 min	Original fatty acids 78% remaining in hydrochar, total fatty acid recovery 49%, the yield and phosphorus recovery more than 70%	[189]
Dairy cattle manure	Novel hydrochars co-activated by thiourea and Fe(NO_3_)_3_ via one-pot and two-stage schemes	Maximum adsorption capacity of As (V) 98.74 mg g^−1^, distribution coefficient 85.67 mg (g μM) ^−1^	[190]
Waste cotton	250 °C for 3 h, then modified with β- cyclodextrin	Adsorption of hydrothermal biochar for Pb^2+^ 50.44 mg g^−1^, adsorption of hydrothermal biochar for Cd^2+^ 33.77 mg g^−1^, removal efficiency of Pb^2+^ in soil 92.87%, removal efficiency of Cd^2+^ in soil 86.19%	[191]
Apple bagasse	Hydrothermal treatment at 180 and 230 °C for 2 and 4 h	Energy densification 1.3–1.6 with 80%-93% of C recovering, carbonaceous materials for CO_2_ neutral fuel (30 MJ Kg^−1^) and soil improver	[192]
Wheat straw and poplar sawdust	Hydrothermal carbonization at 260 °C, then treated by anaerobic fermentation (AF)	Maximum adsorption capacity of modified wheat straw hydrochar increased by 3.1 times (19.87 mg g^−1^), maximum adsorption capacity of modified poplar sawchip hydrochar increased by 3.4 times (16.68 mg g^−1^)	[193]
Corn straw (CS) and eucalyptus leaves	Prepare modified artificial humic acid, then colloid-like magnetic biochar was fabricated in one step through hydrothermal reaction	Cd^2+^ removal capacity 169.68 mg/g with rich functional groups, great dispersibility, oxidation resistance	[194]

Hydrochar from simulated food waste retained up to 78% of the original fatty acids and accomplished a total fatty acid recovery of 49%. Under mild conditions, the yield and phosphorus recovery of aqueous phase products were higher than 70% [189]. The optimum conditions for tetracycline removal were 598.63 mg L^−1^ of CuFeO_2_/BC-1.0, 57.63 mM of H_2_O_2_ and pH of 6.27. The maximum adsorption capacity of As (V) and distribution coefficient were respectively 98.74 mg g^−1^ and 85.67 mg (g μM) ^−1^ in a study on novel hydrochars co-activated by thiourea and Fe(NO_3_)_3_ via one-pot and two-stage schemes for the processing of dairy cattle manure [190]. Adsorption of hydrothermal biochar derived from waste cotton for Pb^2+^ and Cd^2+^ reached 50.44 and 33.77 mg g^−1^, respectively, while the removal efficiency of Pb^2+^ and Cd^2+^ in soil reached 92.87 and 86.19%, respectively [191]. HTC of apple bagasse led to energy densification of 1.3–1.6 with 80–93% of C recovering and generated stable carbonaceous solids to be applied as CO_2_ neutral fuel (30 MJ Kg^−1^) and soil improver [192].

In a study engrossed on HTC and anaerobic fermentation of wheat straw and poplar sawdust, the maximum adsorption capacity of modified wheat straw hydrochar and modified poplar sawchip hydrochar increased by 3.1 times (19.87 mg g^−1^) and 3.4 times (16.68 mg g^−1^), respectively [193]. The 3DPC exhibited abundant sp^3^ defects and micropores with a surface area of 855.12 m^2^ g^−1^, while the as-assembled OCMSs and 3DPC potassium ion hybrid capacitor presented an energy of 140.7 Wh Kg^−1^ at 643.8 W Kg^−1^ with a recycling circle of more than 8500 times. Colloid-like magnetic biochar (Col-L-MBC) was fabricated in one step through hydrothermal process of corn straw and eucalyptus leaves in presence of modified artificial humic acid (A-HA) [194]. Col-L-MBC has rich functional groups, great dispersibility, oxidation resistance, and considerable Cd^2+^ removal capacity (169.68 mg g^−1^).

Hydrochars derived from HTC present gainful characteristics such as large surface area, high porosity, and multiple surface functional groups, which are beneficial to be used in environmental remediation. This process is to be relatively straightforward, as only one target solid product, and do not need extra procedures to collect other products. However, the HTC technology is still under developing. It is a promising technology but still needs further detailed investigation for its potential applications in energy and environmental sustainability.

### Pyrolytic Carbonization

Pyrolysis is a common thermochemical process to treat biomass, which can be rummage-sale in the production of syngas, bio-oil, biochar, and other products. Product yields mainly depend on pyrolysis conditions. According to pyrolysis time and temperature, pyrolysis can be classified into "slow" pyrolysis and "fast" pyrolysis. Different feedstock and approaches in pyrolytic carbonization (PC) are summarized in Table 7. Pyrolytic carbonization of bamboo waste was carried out in a fixed-bed system, achieving a maximum specific surface area of 1351.13 m^2^ g^−1^ [195]. This process also developed high porosity and facilitated oxygen groups evolution into more stable –OH, –CO, and –COOH groups. In a study on pyrolytic carbonization of maple leaf, the maximum surface area reached 191.1 m^2^ g^−1^, and calcite crystal and hydrophobicity increased significantly [196]. The adsorption capacity was 407.3 mg g^−1^ for the removal of tetracycline by maple leaf derived biochar. Specific surface area, pore diameter and total pore volume of biochar reached 594 m^2^ g^−1^, 3.1 nm and 0.93 cm^3^ g^−1^, respectively, in conversion of shrimp shell under 800 °C, 2 h reaction time, N_2_ atmosphere and 5 °C min^−1^ [197].

**Table 7:** Different feedstock and approaches in pyrolytic carbonization

Feedstock	Approach	Product characteristics	References
Bamboo waste	KOH/biomass ratios 1:8 to 1:1, temperatures 400–800 °C	Maximum specific surface area 1351.13 m^2^ g^−1^, developed porosity, oxygen groups into more stable -OH, –CO and –COOH groups	[195]
Maple leaf	750 °C under a constant nitrogen atmosphere for 2 h	Maximum surface area 191.1 m^2^ g^−1^, calcite crystal and hydrophobicity increased significantly, the adsorption capacity of tetracycline 407.3 mg g^−1^	[196]
Shrimp shell (PSS-bio)	800 °C for 2 h under N_2_ atmosphere with the heating rate of 5 °C min^−1^	Specific surface area of biochar 594 m^2^ g^−1^, pore diameter 3.1 nm, total pore volume 0.93 cm^3^ g^−1^	[197]
Renewable biomass (e.g., rice husk, saw dust, corn stalk)	Via a fast pyrolysis at 500 °C coupled with atmospheric distillation process	High heating values of the as-prepared bio-coals 25.4 to 28.2 MJ Kg^−1^ (comparable to that of the commercial coals)	[198]
Vine shoot, crystalline cellulose	Via pyrolysis of chemical zinc chloride modified raw materials in carbon dioxide atmosphere for the preparation of lead-based catalysts	Specific surface area of biochar 2867 m^2^ g^−1^, pore diameter 2.7 nm, pore volume 1.020 cm^3^ g^−1^, catalyst selectivity toward the dehydrogenation reaction 100%	[199]
Sewage sludge	600 °C for 4 h under the N_2_ environment, KOH pretreatment (flaked formed) and combined treatment	Specific surface area of biochar 264.1 m^2^ g^−1^, total pore volume 0.449 cm^3^ g^−1^ with superior peroxymonosulfate activation and degradation capacity of organic pollutants	[200]
Biomass wastes (lignin, cellulose, wheat straw and sawdust, etc.)	Utilizing waste pyrolysis gases and waste heat to prepare high-quality three-dimensional graphene foams (3DGFs)	3DGFs consist mainly of approximately 95% C, 3% O, and 1% H with a mass density of 2.4–3.0 mg cm^−3^	[201]
Spent mushroom substrate (SMS)	At 600/300 °C for 4 h under CO_2_/N_2_	600 °C and CO_2_ produced higher levels of aromaticity, ash, S_BET_ and porosity (S_BET_ increased 4.19-fold, total pore volume increased 9.6-fold); 300 °C and N_2_ produced more oxygen-containing functional groups	[202]
Waste bean dregs	900 °C for 2 h under N_2_ atmosphere	Specific surface area of biochar 3000 m^2^ g^−1^, catalytic activity for bisphenol A 1,358.4 mg g^−1^	[203]
Teak sawdust	400–700 °C, nitrogen flow rate of 150–250 mL min^−1^ for 60 min	The yield of teak sawdust pyrolytic biochar 27.4% at 600 °C, BET surface area of biochar 253.66 m^2^ g^−1^ with high calorific value	[204]

Renewable biomass (e.g., rice husk, saw dust, corn stalk, etc.) were rehabilitated via fast pyrolysis at 500 °C coupled with atmospheric distillation process [198]. The results showed that the HHV of the as-prepared bio-coals from the representative biomass were within 25.4–28.2 MJ Kg^−1^, which are comparable to that of the commercial coals. Vine shoot and crystalline cellulose derived biochar was prepared by pyrolysis of chemical zinc chloride modified raw materials in carbon dioxide atmosphere for the preparation of lead-based catalysts [199]. The specific surface area, pore diameter and pore volume of biochar reached 2867 m^2^ g^−1^, 2.7 nm, and 1.020 cm^3^ g^−1^, respectively, while catalyst selectivity toward the dehydrogenation reaction reached 100%. KOH treatment significantly increased the specific surface area of sewage sludge-derived biochar (264.1 m^2^ g^−1^), enhanced the pore structure (total pore volume 0.449 cm^3^ g^−1^), and had superior peroxymonosulfate activation and degradation capacity of organic pollutants [200].

A study engrossed on utilizing waste pyrolysis gases and waste heat to prepare high-quality three-dimensional graphene foams (3DGFs) [201]. The results demonstrated that biomass wastes derived 3DGFs consist mainly of approximately 95% C, 3% O, and 1% H. 3DGFs prepared from biomass pyrolysis gas were ultra-light with a mass density of 2.4–3.0 mg cm^−1^. Spent mushroom substrate was investigated at 600/300 ℃ for 4 h under CO_2_/N_2_ [202]. The results illustrated that 600 °C and CO_2_ produced higher levels of aromaticity, ash, BET surface area and porosity (BET surface area and total pore volume increased 4.19- and 9.60-fold, respectively), while 300 °C and N_2_ produced more oxygen-containing functional groups, such as hydroxyl, carboxyl, and carboxyl groups. The waste bean dregs derived biochar had extremely high specific surface area (BET surface area, > 3000 m^2^ g^−1^) [203]. Besides, the biochar showed good catalytic activity for bisphenol A (BPA, 1358.4 mg g^−1^). The yield of teak sawdust pyrolytic biochar was 27.4% at temperature of 600 °C [204]. The BET surface area of biochar was 253 m^2^ g^−1^ with high calorific value, which can be used as solid fuel and suitable for waste stream purification.

A comparative insight was demonstrated on the fuel performance of chars derived from the two pretreatment methods of HTC and PC [205, 206]. The key parameters including VM, the solid yield, HHV, the N content reduction, and char reactivity can be adopted to well evaluate the qualities of solid biofuel produced from different processes of HTC and PC, respectively. Sustainable solid fuel production was investigated via HTC technology using municipal solid wastes and sewage wastes [207]. Since the hydrothermal method is not limited by the moisture content of raw materials, it can be used for the carbonization of HALUB biomass with high moisture content, such as sewage sludge, aquatic plants, food, and agricultural waste, which are difficult to be processed by pyrolysis or gasification system.

## Advanced Technologies of HALUB Conversion and Management

IN this section, we focused on advanced technologies of nano-catalysis and machine learning in the field of HALUB conversion and management.

### Nano-catalytic Technologies

Nano-catalytic technologies have attracted courtesy owing to their advantages of environmental sustainability, high efficiency, and low emissions for carbon neutrality, especially their wide applications in the production of energy and materials [208, 209]. A summary on the nano-catalytic conversion biomass to energy and material is tabulated in Table 8. Graphitic carbon supported Co catalyst was applied for catalytic steam reforming of tar [210]. The Co_0.1_/oxidized Shengli lignite char catalyst maintained a stable toluene conversion of 85% during the 30-h test of the steam reforming of tar. It can efficiently remove tar and simultaneously convert tar into high-value gases. Lignocellulosic biomass was converted over in-situ self-regenerable Fe/Fe_3_C-Mo_2_C-CNF catalyst [211]. Average hydrodeoxygenation conversion and gas yield of 45–50% showed that lignocellulosic biomass could be largely converted to hydrogen rich syngas through addition of external CH_4_ and CO_2_. Catalytic biomass tar cracking was studied over highly dispersed FeNi alloy catalysts embedded in graphitic carbon (BC-FeNi) [212]. The conversion rate of tar was 95.8% in presence of 1.01% metal load nano-catalyst, which could effectively convert tar compounds into syngas at 800 °C.

**Table 8:** Nano-catalytic conversion biomass to energy and materials

Feedstock	Nano-catalyst	Approach	Product application	References
Tar	Graphitic carbon supported Co catalyst	Catalytic steam reforming of tar	Efficiently tar removal and high-value gases production	[210]
Lignocellulosic biomass	In-situ self-regenerable Fe/Fe_3_C-Mo_2_C-CNF catalyst	Fe-Mo_2_C-CNF catalysts for CH_4_-CO_2_ assisted biomass reforming	Value-added chemicals such as ammonia, olefin and methanol, or as gas turbine fuel	[211]
Biomass tar	Highly dispersed FeNi alloy catalysts embedded in graphitic carbon (BC-FeNi)	Catalytic biomass tar cracking	Clean energy source	[212]
Biomass-derived monosaccharides	Zinc sulfide and bismuth sulfide photocomposite catalyst (ZnS@Bi_2_S_3_) nanosheets	ZnS@Bi_2_S_3_ nanosheets as a composite photocatalyst to selectively oxidize xylose to xylonic acid	Chelating agent, dispersant, clarifier, pH regulator, antibiotic, and health enhancer	[213]
Waste chicken eggshells	CaO-TiO_2_ nano-catalyst	Via transesterification reaction of glycerol with DMC in a reflux condensation process	Food, medicine, new energy, and other fields	[215]
Ethylene glycol	Alumina supported Ni-Pt bimetallic catalysts	Steam reforming	Alternative to fossil fuels	[216]
Seed oil	Trimetallic based montmorillonite nano-catalyst	Via two-step transesterification reaction	Diesel engine	[217]
Algal oil	Heterogeneous nano-catalyst, Ca(OCH_3_)_2_	The transesterification of *N. oculata* algae	Biodiesel	[218]
Biomass-derived alcohols	Cobalt/nitrogen-doped carbon catalyst	Oxidative esterification of biomass-derived alcohols	Alternative monomer for the generation of bio-derived polymers	[219]
Vanillyl alcohol, vanillin and lignin	Pd/Ru metal supported graphene oxide nano-catalysts	Hydrodeoxygenation (HDO) of lignin monomer molecules- vanillyl alcohol and vanillin	Vanillin and *p*-cresol	[223]

A study engrossed on conversion of biomass-derived monosaccharides via ZnS@Bi_2_S_3_ nano-catalyst was conducted via a facile one-pot synthetic route to selectively oxidize xylose to xylonic acid, achieving a conversion rate of 91.6% and d-xylonic acid yield of 74.2% [213, 214]. CaO–TiO_2_ nano-catalyst was applied in co-transesterification reaction of waste chicken eggshells, glycerol, and glycerol carbonate in a reflux condensation process. The results showed that CaO/TiO_2_ provided 99.3% conversion of glycerol with 93.7% of glycerol carbonate yield, it can be used in food, medicine, new energy and other fields [215]. Steam reforming was applied to convert ethylene glycol over alumina supported Ni-Pt bimetallic catalysts [216]. The highest ethylene glycol conversion, H_2_ selectivity and yield reached 60, 45 and 27%, respectively. Seed oil was converted via two-step transesterification reaction over trimetallic based montmorillonite nano-catalyst [217]. The density value, acidity value, flash point value, turbidity point and pour point of the biodiesel obtained were all within the standard range, so it could be used in diesel engine.

Algal oil was progressed via transesterification of *N. oculata* algae in the presence of a heterogeneous nano-catalyst and Ca(OCH_3_)_2_ [218]. This process resulted in a 34.6% reduction in carbon dioxide emissions and a biodiesel purity of 91.55%, which encouraged the production of biodiesel from algal oil. Oxidative esterification of biomass-derived alcohols was carried out over cobalt loaded on nitrogen-doped carbon nano-catalyst [219]. The results showed a 96% quantitative conversion of HMF and 92% selectivity of FDMC at 80 °C. Hydrodeoxygenation (HDO) of lignin monomer molecules-vanillyl alcohol and vanillin was conducted over Pd/Ru metal supported graphene oxide nano-catalysts [220]. This method was directly applied to the chemical transformation of phenolic intermediates generated by photocatalytic cracking to obtain vanillin and *p*-cresol, which can be formed as a potential biofuel in the future [221]. To summarize, nano-catalytic technologies are of high importance in the efficient and complete conversion of biomass-derived feedstocks, biowaste, and bio-oil, thus has a promising application in the conversion of HALUB [222].

### Machine Learning

**Fig. 8:**
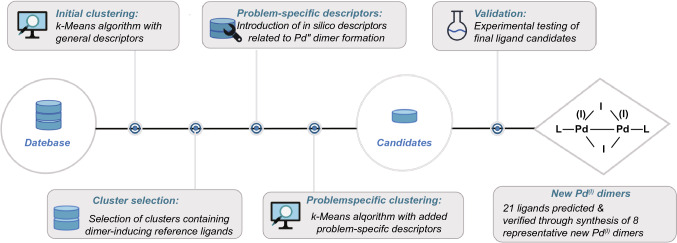
Application of machine learning workflow to predict new Pd^(I)^ dimers

In the twenty-first century, the combination of big data and artificial intelligence (AI) is known as the fourth paradigm of science [224] and the fourth industrial revolution [225]. Among them, machine learning (ML), as an essential data analysis technology, has been widely concerned by all walks of life and achieved explosive development in the past decade. ML is a collection of advanced data analysis methods with statistical algorithms as the core, which can acquire and integrate knowledge independently and has high prediction accuracy. In recent years, ML has been successfully applied to cutting-edge work in the fields of medicine [226], physical science [227], biology [228], earth system science [229], and material science [230]. ML is suitable for solving problems that are difficult to be solved by traditional methods for challenging problems, such as those involving a large number of combinatorial spaces or nonlinear processes. This method saves time, reduces labor and resource consumption, and is often accompanied by new discoveries. For example, in a recent study, unsupervised machine learning successful identification of the ligand, similarity between forecast algorithm and some phosphine ligands is verified by experiment, and synthesized eight had not been reported before air stable Pd^(I)^ dimers [231]. Figure 8 shows the application of machine learning workflow forecast form and identify new Pd^(I)^ dimers process.

At present-days, ML has attracted more and more devotion in the field of transformation of biological resources and has been extensively used to predict the transformation behavior of biomass through pyrolysis, gasification, hydrothermal and co-conversion. For example, traditional hydrothermal liquefaction experimental method to produce bio-oil requires a lot of time and manpower in order to obtain bio-oil with high yield and low nitrogen content. Therefore, ML algorithm is used to assist bio-oil production. After experimental verification, the actual yield of bio-oil is 54.30%, the content of n is 2.60%, and the energy recovery rate is 75.42%. The results are not different from the bio-oil yield (58.11%), nitrogen content (3.37%) and energy recovery rate (80.32%) optimized by the model, ML provides a new idea and strategy for accelerating the production of high-quality engineering bio-oil [232]. In another study of lignocellulosic biomass pyrolysis, the prediction model of biochar yield and carbon content using machine learning was successfully developed. The model can accurately predict biochar yield and carbon content according to biomass characteristics and pyrolysis conditions. Among them, the relative contribution of pyrolysis conditions to yield (65%) and carbon content (53%) is higher than that of biomass characteristics, which can help us understand the biomass pyrolysis process and provide new ideas for improving biochar yield and carbon content [233]. Biomass gasification is a promising power generation process. Predicting this process is conducive to obtaining the best products. Before the application of ML attracted attention, scientists proposed various kinetic models and equilibrium models, but the assumptions in these models greatly reduced the actual availability and consistency. A study based on ML prediction predicted the CH_4_, H_2_, CO, CO_2_ and HHV outputs of downdraft biomass gasification process. The results showed that most of the outputs reached R_2_ > 0.9 and were superior to traditional modeling methods in accuracy. These models can be used in simulation environment, microcontroller circuit or practical application [234].

**Fig. 9:**
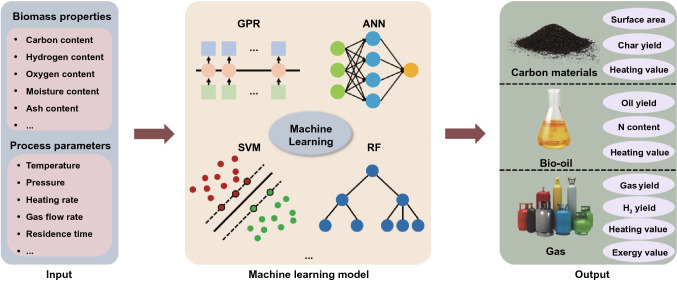
Application of machine learning in modeling of biomass thermochemical conversions. GPR: Gaussian process regression; SVM: support vector machine; RF: random forest (RF); ANN: artificial neural network

Compared to traditional mechanism models, data-driven ML models can learn and recognize nonlinear relationships between the output and input parameters [235]. The widely used ML models include Gaussian process regression (GPR), support vector machine (SVM), random forest (RF), and artificial neural network (ANN) models (Fig. 9) [236].

The ANN model has been widely used to forecast and optimize system outputs in a short CPU time, which simulates the human brain in terms of mathematical functions [237]. An ANN includes an input layer, a number of hidden layers, and an output layer. Multilayer network structures and feed-forward ANNs with the backpropagation method is strong and widely used [238]. ANN models are particularly powerful for solving the extensive problems in science and engineering and have been used by many researchers in complex biomass thermochemical conversion processes [238]. The ANN model as a machine learning method was used to investigate the exergy value of the syngas. The Levenberg–Marquardt algorithm was used to train the ANN model. The oxygen, hydrogen, and carbon contents of 16 different types of biomass, gasification temperature, steam and fuel flow rates were the input parameters. The hydrogen percentage in the syngas and the exergy value of the syngas were predicted accurately [239]. In another study, ANN models were also applied in the steam gasification of biomass to predict the product yield. It has been suggested that ANN models perform better than traditional regression models [240].

In a bubbling fluidized bed reactor, the effect of bed materials was included in the input of the ANN model to predict the gas composition (H_2_, CO, CO_2_, and CH_4_) and gas yield. The training of the networks was carried out with feed and cascade forward back propagation networks with one and two hidden layers and with Levenberg–Marquardt and Bayesian regulation learning algorithms. It has been reported that the output matched well with the experimental data, with an R^2^ higher than 0.94. These results indicated that the ANN model is a powerful tool to help the design and operation of the reactor, as well as the control of pollutants [237]. Large-scale experiments are usually expensive and energy intensive. Therefore, neural network models are beneficial for use in industrial-level plants. In a large-scale biomass gasification plant, the ANN model showed good capability to predict the biomass gasification process [241].

For the supercritical gasification (SCWG) of biomass, four ML models were applied to predict the H_2_ production. The results suggested that the RF model exceeded the others (GPR, ANN, and SVM models) with an* R*^2^ of 0.9782. The RF model was combined with feature importance and partial dependence analysis to visually present the relative important and average partial relationship between the H_2_ yield and the input parameters. This study indicated that ML is useful for predicting H_2_ production from the SCWG of biomass [242].

The yield and quality of bio-oil are affected by many factors, such as biomass feedstock type (biomass type, particle size, pretreatment), operation conditions (temperature, pressure, heating rate, reactor type), and catalysts [243]. Numerous experiments have been performed to obtain the bio-oil from biomass pyrolysis under different conditions. Therefore, it is meaningful to develop a model to predict the bio-oil yield and quality from the above-mentioned factors. The ANN and SVM were used to predict the production distribution and bio-oil heating value (HV) of biomass pyrolysis. Correlated samples of biomass pyrolysis were collected as the data set. It was shown that both ANN and SVM can predict the product yield and HV of bio-oil. The SVM model could make the prediction better compared to the ANN model [244].

ML has also been used in the prediction of bio-oil production from hydrothermal liquefaction of biomass with the input parameters of biomass elemental composition, process parameters, and solvents. RF performed the best with the *R*^2^ of 0.80 for the prediction of bio-oil yield, nitrogen in oil, and oil energy recovery. Therefore, the ML could be used to guide the experiments to produce bio-oil with a low N content [245].

Some progress has also been made in the application of ML in the synthesis of carbon materials from biomass. A deep neural network (DNN) model was used to predict the fuel properties and carbon capture and storage (CCS) stability of hydrochar with the *R*^2^ of 0.91. ML has revealed that both the fuel elemental composition of biomass and temperature are key factors for the characteristics of hydrochar [246].

Although ML plays a chief protagonist in the transformation of biological resources, ML-assisted prediction is still in the initial stage of development, and there are few related research contents and lack of comparison between different models, which will have great application potential in the future.

### Microfluidics

Microalgae have been considered as promising alternatives for the production of biodiesel as an alternative to fossil fuels [247]. However, their application is limited by the fact that the production costs for large-scale derivation of biofuels and bioproducts from microalgae are still much higher than economic feasibility. A great deal of work has been done to overcome this limitation. Typically, neutral lipids occur as triacylglycerols (TAG), which are synthesized in algae through an ester exchange process using an intermittent reactor. However, this process can lead to non-homogeneous conditions and subsequent inefficient cell growth, resulting in a potential decrease in total biodiesel yield.

**Fig. 10:**
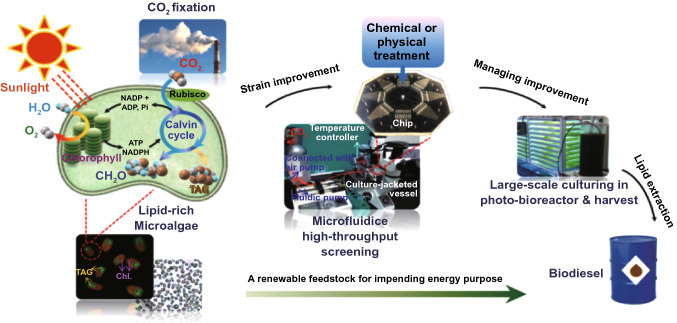
Schematic illustration of all steps of the microalgae-based biofuel production pipeline and how the developed GNT-Microfluidic chemostatic bioreactor system work. Algal cell structure serves goals of biodiesel production through the photosynthetic carbon fixation pathways the Calvin-Benson cycle

To overcome this paradox, a "two-stage culture" strategy in bioreactors has been explored to improve the yield of microalgal lipids [248]. However, a detailed study of the stress response of lipid-rich microalgae in continuous culture may provide mutual benefits for the one-step production of biomass and lipids, and this strategy may be more suitable for large-scale production [249]. However, currently available bioreactor systems are not very efficient and suffer from problems such as high microalgal adhesion disrupting biological properties and unsuitability for high-throughput screening applications. Zheng et al. [250] proposed a microfluidic chemical bioreactor (Fig. 10) that provides low bioadhesive cultures of algae in a synergistic environment of gas, nutrients and temperature (GNT) with high-throughput screening capabilities. In addition, the core chip of this reactor is a high-throughput microfluidic bioreactor array capable of simultaneously studying the effects of 64 different nutrient conditions on microalgal growth and oil production, overcoming the limitations of conventional culture systems.

## Applications of Sustainable Natural Biomaterials

Abundant materials, ideas, possibilities, and sustainable solutions were provided by Mother Nature. Nature-derived and Nature-inspired materials shine the glory of intelligence for a large variety of sustainable energy, environment, and especially biomedical applications due to their unique advantages, including the excellent biocompatibility, biodegradability, vast abundance, low-cost, diverse functionalities, and beyond [251–259].

### Multifunctional Applications

**Fig. 11:**
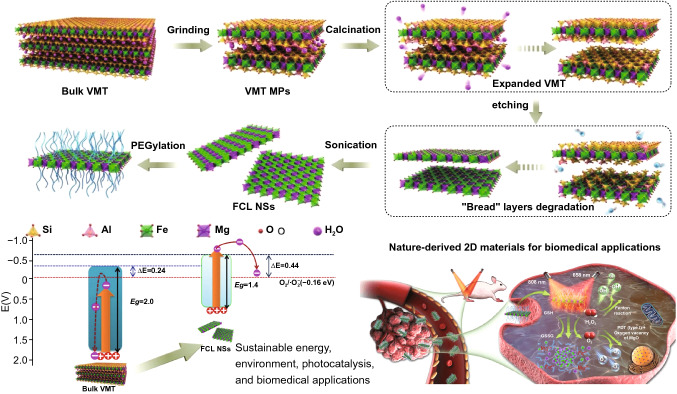
Nature-derived 2D materials fabrication and PEGylation (reprint with permission from Nature Publishing Group [263])

Many stunning advanced materials have been developed via bioresource upgrade. One great example is the two-dimensional (2D) materials. 2D nanosheets were upgraded from FDA-approved compositions from clays by Harvard scientists [260]. Those 2D nanosheet derived from natural clay has been demonstrated for diverse applications, including cancer therapy. Clays, also known as phyllosilicate minerals, are fundamentally composed of tetrahedral silicon (SiO_2_) and/or aluminum oxide (Al_2_O_3_) crystal structures. To upgrade the resources of clays, researchers have proposed a universal exfoliation method that is able to intelligently “capture” the ultrathin, biocompatible, and functional core layers (FCLs: MgO and Fe_2_O_3_, both are FDA-approved). These materials are sandwiched between two identical tetrahedral layers (SiO_2_ and Al_2_O_3_) from 2:1 aluminosilicate (vermiculite (VMT), biotite, flogopite, illite, etc.). The processes to upgrade the natural clays include a combination of ball-grinding, calcination, etching, and sonication. The above-mentioned NSs have an average thickness of 2.7 nm and a size of 110 nm, respectively. For its applications in nanomedicines for in vivo therapy, physiological stability and dispersibility are important indicators. Thus, the FCL nanosheets were further modified by positively charged PEG-NH2. The average thickness of FCL-PEG NSs increased to 6 nm, which confirmed the successful PEG-NH2 functionalization. Meanwhile, the average size of FCL-PEG NSs decreased to 105 nm, which is a result of the use of bath sonication to break down FCL NSs during PEGylation. Given the fact that the FDA-approved MgO and Fe_2_O_3_ are widely used in the clinic for the treatment of stomach diseases and iron deficiency respectively, the innovative grown layers are biocompatible and potentially highly benefit both the basic science and translational medicine. Additionally, both in vivo and in vitro toxicity studies were conducted to further confirm and highlight the excellent biocompatibility of the obtained FCL-PEG NSs. This 2D nanosheet further specifically pioneers their application in cancer theranostics as a demonstration of proof-of-concept, which also shows their potential as a prelude to the future extensive studies of 2D NSs (Fig. 11).

The FCL-PEG NSs upgraded from natural clay had a strong ability to modulate tumor microenvironment (TME) via catalyzing H_2_O_2_ to produce O_2_, while consumes GSH due to the existence Fe^3+^ in FCL-PEG NSs, which could relieve hypoxia and diminish the antioxidant capability of the tumor.

The NSs upgraded from natural clay possess a tunable and appropriate electron band structure with the bandgap decreased from 2.0 to 1.4 eV and the conductive band increased from –0.4 to –0.6 eV. This result endows them a huge potential in energy, catalysis, and biomedicine. By taking advantages of the narrowed band gap and improved *ΔE* between the conductive band of FCL-PEG NSs and E0 of O_2_/·O_2_^−^, effective electron–hole separation of FCL NSs has been explained under 658 nm laser irradiation, which elevated the ·O_2_^−^ generation from O_2_ with a high photodynamic therapy (PDT) efficacy.

Another good example is naturally upgraded fluorosurfactant. It has been widely used in various areas, including the fundamental research and the industrial applications. Fluorosurfactant-stabilized droplets can be generated by microfluidics, which are widely used as pico- to nanoliter volume reactors for both chemistry and biology. However, current available surfactants are not able to completely prevent the inter-droplet transfer of small organic molecules that encapsulated or produced inside the droplets. In addition, most microdroplets typically coalesce at temperatures higher than 80 °C. Thus, the usages of fluorosurfactant-stabilized droplets for ultrahigh-throughput combinatory drug screening and polymerase chain reaction (PCR) have been limited. Bioresource upgrade has been employed into designing surfactants that form robust microdroplets with improved stability and capability to prevent inter-droplet transferring. To upgrade surfactant, a panel of dendritic oligo-glycerol-based surfactants have been produced [261]. The authors further elaborated a high degree of inter-and intramolecular hydrogen bonding, as well as the dendritic architecture, which guarantees high droplet stability. The good stability further benefits PCR thermal cycling via minimizing the inter-droplet transfer of the water-soluble fluorescent dye sodium fluorescein salt and the drug doxycycline.

The bioresource upgrade can be pragmatic to biomedical therapy. For example, the ROS can be upgraded. A sublethal level of ROS sustains cell proliferation, differentiation and promotes tumor metastasis, while a drastic ROS burst directly induces apoptosis. The biological derived nanomaterials have been applied for ROS modulation. The surface-oxidized arsenene nanosheets (As/As_*x*_O_*y*_ NSs) with type II heterojunction are upgraded for efficient ·O_2_^−^ and O_2_ production [262]. The upgraded biomaterials also consume glutathione through prolonging the lifetime of photo-excited electron–hole pairs. Additionally, the portion of As_*x*_O_*y*_ is not only able to catalyze a Fenton-like reaction, but also generate ·OH and O_2_ from H_2_O_2_. At the same time, As/As_*x*_O_*y*_ NSs will be inactively main antioxidants to prevent the cytotoxicity of ROS. The As/As_*x*_O_*y*_ NSs can further be upgraded by coating polydopamine (PDA) and cancer cell membrane. The further upgraded As/As_*x*_O_*y*_ NSs provides as an intelligent therapy system with active tumor targeting and long-term blood circulation. More importantly, As_*x*_O_*y*_ can be further upgraded to As/As_*x*_O_*y*_@PDA@M NSs for imaging-guided non-invasive and real-time nanomedicine for cancer therapy.

The natural bioresource can also be progressed to wearable biomedical and theranotic devices. A good example is a non-printed integrated-circuit textile (NIT), which is built by fibers via interlacing nodes and waving into a deformable textile integrated circuit [263]. The upgraded non-printed integrated-circuit textile builds electrochemical gates. In the device, the fiber-woven-type transistors have been demonstrated with superior bending or stretching capability. This non-printed integrated-circuit textile was woven into a fiber-type sweat sensor with strain and light sensors, providing an intelligent wearable device for simultaneously monitoring body health and the environment. The woven circuit textile can be completely self-powered with a photo-rechargeable energy textile for both wireless biomedical monitoring and early warning. As a demonstration of proof of concept, the non-printed integrated-circuit textile could be used as a 24/7 private AI “nurse” for routine healthcare, healthy monitoring, or emergencies such as hypoglycemia, metabolic alkalosis, and even COVID-19 patient care.

In addition to the natural materials, the living cells can also be upgraded for biomedical applications. For example, a helical-shaped cyanobacterium, Spirulina platensis (SP), can be upgraded by loading curcumin (SP@Curcumin) [123]. The upgraded microalgal biomass can be employed to treat colon cancer and colitis, two different types of gastrointestinal diseases. SP@Curcumin encapsulated in microalga was used for the combined chemo- and radiotherapy, resulting in inhibition of tumor progression and radioprotection by scavenging reactive oxygen species that generated by the high dose of X-ray radiation. The upgraded microalga further reduces the production of proinflammatory cytokines to inhibit inflammation against colitis. Based on the above-mentioned parameters of the helical microalgae, it has been further upgraded for the protection of the whole small intestine from radiation-induced intestinal injury in the radiotherapy of gastrointestinal solid tumor [264].

**Fig. 12:**
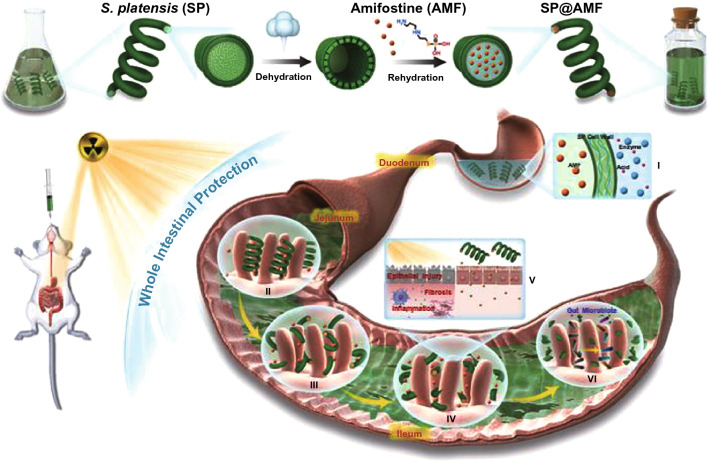
Schematic illustration of upgradation of SP@AMF and its radioprotective mechanisms. I. Schematic illustration of the mechanism how SP protects AMF from gastric destruction. II–IV. Schematic illustration of the controlled releases AMF form the upgraded SP@AMF when traveling along the small intestine. V. Schematic illustration of the protection of SP@AMF from radiation-induced epithelial injury, inflammation, and fibrosis. VI. Schematic illustration of SP@AMF for maintaining the health of gut microbiota [267]. (Reprint with permission from Nature Publishing Group)

Despite of the recent advances of selective radioprotector for healthy tissues such as Amifostine (AMF), its applicant to intestinal radioprotection has been limited due to the harsh microenvironment of gastrointestine and rapid refreshment. A microalga carrying AMF, SP@AMF, has been constructed for oral delivery for radioprotection. The SP@AMF exhibit superior drug accumulation and radioprotection in the whole organ as compared to free AMF and its enteric capsule. More significantly, the SP@AMF prevented the radiation-induced early and delayed intestine injury, which resulted in prolonging the survival without influencing the tumor regression. To upgrade the microalga SP, SP was first lyophilized (dehydration) followed by incubated with a solution of AMF. The AMF will flow into the dehydrated SP during its extra flow-mediated drug loading and the rehydration process (Fig. 12). In addition, the upgraded microalga can be employed for automatic marine pollutants monitoring. The motion of algae can be set as a signal for bioassay sensor of marine pollutants [264]. The results demonstrated by Han et al. showed that *Platymonas subcordiformis* as a sensitive and robust bioreporter when encapsulated in digital microfluidic systems. The microfluidic device can be extended to a gradient generator, which enable screening microalgae behaviors in a simple and cost-/time-/space-saving way [265]. This study conducted by Zheng et al. showed more opportunity for the upgraded microalgae as biosensors and monitors.

### Sustainable Living Materials

Granting natural biomaterials such as wood and cotton have been extensively used in life and industry, their characteristics are limited by evolutionary selection and are still not ideal. Sustainable living materials (SLMs) integrate biological and abiotic components and have the advantages of both synthetic and natural materials, which can meet this limitation. As a rapidly developing emerging field, SLMs aim to summarize the ideal characteristics of natural biomaterials and create new materials with activity and responsiveness by using genetically engineered organisms [266, 267]. A variety of organisms, such as bacteria, fungi, and algae, have been incorporated into materials such as concrete and hydrogels, and have broad application prospects in the fields of daily life, construction, and medical treatment. For example, a living component of silica material composed of a self-assembled protein scaffold has been prepared. The obtained SLM can respond to external stimuli and can be regenerated from cells containing silicon material and incorporate new functions. The SLM can be utilized as a self-healing material and can be applied to coatings and gypsum [268].

**Fig. 13:**
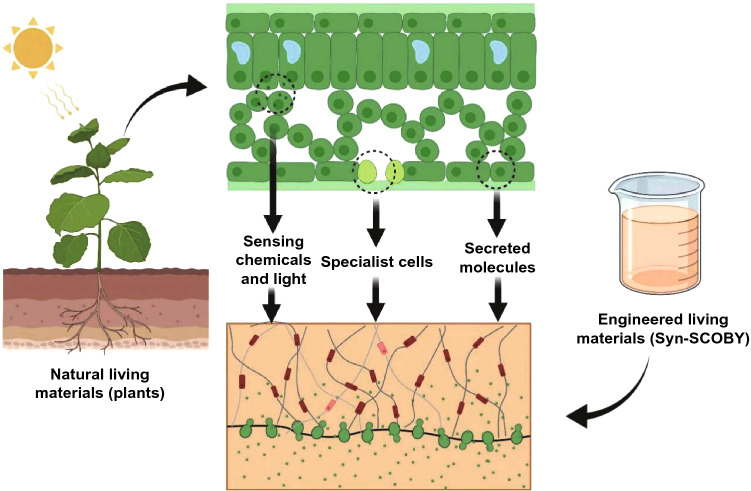
Schematic diagram of analogies between natural living materials (plants) and Syn-SCOBY materials for engineered living

Compared with plant cellulose, bacterial cellulose (BC), as a high value-added biomass material, has some additional and unique properties, such as a high degree of polymerization, high crystallinity, high purity, and biocompatibility. Because BC can obtain a high yield in a short period of time, it has recently become a promising material to produce SLMs. However, due to the lack of genetic tools and knowledge, the bacteria that produce BC have been reprogrammed to sense external signals. A new SLM system (Syn-SCOBY) has been developed to prepare functional BC-based biomaterials by stable co-culture of Saccharomyces cerevisiae and bacteria. This method creates living materials that can sense and respond to stimuli, and has potential applications in biocatalysts and biosensor [269]. Figure 13 shows the analogy between natural living materials (plants) and engineering living materials. As the material basis and the main force of all life, protein also plays an extremely important role in the design of SLMs. Due to the functional diversity of natural proteins in machinery, electricity, catalysis, magnetism, and biocompatibility, it is possible to reasonably design and direct evolution of proteins, which provides sufficient space for the design and function optimization of elms [270]. For instance, a programmable method to manufacture bio-hybrid semi-IPN (sIPN) has been proposed. The process binds functional proteins through covalent bonds, has good biocompatibility and versatility, and has been proved to successfully protect the microbiota in the intestine from antibiotic-mediated interference [271].

In addition, the cost-effectiveness of mass production of materials and its industrial manufacturing speed limit the feasibility of SLMs in practical application, and there continue to be some challenges in the synthesis process. With the continuous development of SLMs, these new materials will bring more functions and applications and bring great benefits to our life in the near future [272].

### Electrochemical Products

Porous materials equipped from biomass have high specific surface area and porosity, light weight, high mechanical properties and damping properties. These excellent properties make them widely used in aerospace, medical, electrochemical, petrochemical, and other fields [273]. They are also high-quality materials for preparing electrochemical products such as electrode and conductor of supercapacitors. Various feedstocks, e.g., black sesame, wood, pollen, coconut meat, and water hyacinth, have been investigated to produce electrochemical products (Table 9). Microwave treatment, thermal reduction and oxidation, vacuum-assisted filtration and hydrothermal treatment were applied to produce supercapacitors. The supercapacitor presented high performance with a specific capacitance of 403.8 F g^−1^ at 1.0 A g^−1^, cycling stability of 10,000 cycles with 98.2% retention, and energy density of 378.7 Wh Kg^−1^ [274]. Wood cell chamber-reduced graphene oxide was produced via carbonization with a specific capacitance of 288 F g^−1^ and energy density of 36 Wh Kg^−1^ at power density of 3600 W Kg^−1^ [275].

**Table 9:** Conversion biomass to electrochemical products

Feedstock	Approach	Product	Performance	References
Coconut/walnut/bamboo waste	Mechanochemistry/ball milling	lithium–sulfur batteries/medical absorant	Excellent electrochemical performances/better than medicinal charcoal tablets	[274]
Eggshell	Hydrothermal treatment/carbonization	Carbon electrode	Wide potential window, low resistance, high specific capacitance, high cycling capacity	[274]
Coconut meat	Microwave treatment, thermal reduction and oxidation	Supercapacitor	Capacitance 403.8 F g^−1^ at 1.0 A g^−1^, cycling stability 98.2%, energy density 378.7 Wh Kg^−1^	[274]
Wood	Carbonization	Wood cell chamber-reduced graphene oxide@PVA composite material	Specific capacitance 288 F g^−1^, capacitance retention 91%, energy density 36 Wh Kg^−1^, power density 3,600 W Kg^−1^	[275]
Pollen	Template assisted sol–gel methode	Anode	Initial discharge capacity of the full cell 884.1 mAh g^−1^, coulombic efficiency 64.6%	[276]
Tannin (TA)	Vacuum-assisted filtration, hydrothermal treatment	Supercapacitor	Capacitance 548.6 F cm^−3^	[277]
Bamboo pulp	Hydrothermally treated, then directly carbonized	Fibrous film electrode	Specific capacitance of up to 331 F g^−1^ at a current density of 1 Ag^−1^, high energy density of 10.3 Wh Kg^−1^ at a power density of 250 W Kg^−1^	[278]
Water hyacinth biomass	Pyrolysis, followed by HNO_3_ activation	composite sensors	Linearity from 0.74 to 9.82 μmol L^−1^, a limit of detection 0.02 μmol L^−1^, limit of quantification 0.07 μmol L^−1^	[279]
Cellulose nanofibers	Combining 1D CNFs and 2D C-GD nanosheets through a multi-step processing	Triboelectric nanogenerator (TENG)	Short-circuit current (*I*_sc_) 3 μA, open-circuit voltage (*V*_oc_) 38 V, short-circuit transfer charge (*Q*_sc_) 12 nC	[280]
Black sesame	Microwave irradiation	Supercapacitor	Specific capacitance 333.3 F g^−1^, charge transfer resistance (Rct) 0.047 Ω, energy density 3.32 Wh Kg^−1^	[283]
Pine tannin	Pyrolysis	Capacitor electrodes	Maximum electrode capacitance 232 F g^−1^ (at 0.5 A g^−1^), capacitance retention 70% (at 10 A g^−1^)	[284]
Ganoderma lucidum	Chemical self-assembly method	Electrode material	Specific capacitance 176 F g^−1^, rate performance 81.6%, specific surface 893.9 m^2^ g^−1^	[285]
Water hyacinth biomass	Pyrolysis, followed by HNO_3_ activation	Composite sensors	Linearity from 0.74 to 9.82 μmol L^−1^, a limit of detection 0.02 μmol L^−1^, limit of quantification 0.07 μmol L^−1^	[286]
Cellulose nanofibers	Combining 1D CNFs and 2D C-GD nanosheets through a multi-step processing	Triboelectric nanogenerator (TENG)	Short-circuit current (*I*_sc_) 3 μA, open-circuit voltage (*V*_oc_) 38 V, short-circuit transfer charge (*Q*_sc_) 12 nC	[287]
Black sesame	Microwave irradiation	Supercapacitor	Specific capacitance 333.3 F g^−1^, charge transfer resistance (Rct) 0.047 Ω, energy density 3.32 Wh Kg^−1^	[288]
Pine tannin	Pyrolysis	Capacitor electrodes	Maximum electrode capacitance 232 F g^−1^ (at 0.5 A g^−1^), capacitance retention 70% (at 10 A g^−1^)	[289]

The original discharge capacity and the Coulombic efficiency respectively reached 884.1 mAh g^−1^ and 64.6% for a full cell derived from pollen via template assisted sol–gel method [276]. A specific capacitance of 548.6 F cm^−3^ achieved for a supercapacitor derived from tannin via vacuum-assisted filtration, and hydrothermal treatment [277]. Another study investigated fibrous film electrode produced from bamboo pulp by hydrothermal treatment and carbonization [278]. The results exhibit a specific capacitance of up to 331 F g^−1^ at a current density of 1 A g^−1^, and high energy density of 10.3 Wh Kg^−1^ at a power density of 250 W Kg^−1^. Composite sensors derived from water hyacinth presented linearity from 0.74 to 9.82 μmol L^−1^, with a limit of quantification of 0.07 μmol L^−1^ [279]. Triboelectric nanogenerator was produced by combining 1D cellulose nanofibers and 2D nanosheets through a multi-step processing [280]. The results exhibit that a short-circuit current, open-circuit voltage and short-circuit transfer charge reached 3 μA, 38 V, and 12 nC, respectively. These latest advances on conversion biomass to electrochemical products, and multi-system design as demonstrated above, can enable more clean and sustainable electricity generation [281]. It is essential to pin the enhancement on under-researched areas like the engagement of effective catalysts and the development of more stable, efficient, and inexpensive conversion processes [222, 282].

### Micro/Nanomotors

Motion is critical for all different types of lives existing in both macroscopic and micro/nanoscopic realms. Nature has developed smart and high-efficiency biomolecular protein motors through thousands of years of biological evolution and has applied them in numerous biological processes and cellular activities [290, 291]. For instance, bacteria are able to drive themselves forward with the aid of rotary flagella nanomotors. Moreover, linear biomolecular protein motors such as kinesin, myosin and dynein, are able to harvest energy from hydrolyzing adenosine triphosphate (ATP) into adenosine diphosphate (ADP) and phosphate (Pi) molecules for lateral movement along the corresponding tracts. In addition, biological cells are decorated with intelligent biomolecular engines (ATPase) which are demanded to produce biological fuel ATP.

Micro/nanomotors are micro/nanoscale devices, which are capable of converting chemical energy into mechanical force or movement [292, 293]. Evolution bestows biomolecular protein motors with fascinating abilities to harness energy from living ambient for autonomous motion in vivo as described above. Inspired by the fantasy of naturally occurring protein motors, researchers paid great interests into artificial micro/nanomotors in the past decades. In particular, led by pioneering contributions of Sen and Mallouk’s team and Ozin’s group, current work mainly focuses on the exploration of high-efficiency and high-speed artificial micro/nanomotors, which have the abilities to convert chemical energy into autonomous motion [294, 295].

The research of artificial self-propelling micro/nanomotors has rapidly developed in last few decades [296, 297]. Several advanced developments and excellent contributions have been made in this field. Although the bright future of this research field can be expected, some major existing challenges are still remained to be solved. The design, fabrication, control and applications of functional micro/nanomotors require some innovative approaches and ideas to be realized. For example, synthesizing micro/nanomotors with individual functional parts and smartly and precisely controlling motors are still extremely challenging. Hereby, a complete understanding of the physiochemical mechanism is necessary. To realize better control of micro/nanomotors in the future, an industrial level of functional micro/nanomachinery could be achieved. Despite of the significant developments and advances in micro/nanomotors, challenges are still remained to find specific relevant applications, such as biologically compatible fuels, etc.

In terms of implementation, micro/nanomotors have a wide variety of applications, including cargo delivery, water remediation, chemical sensing and biomedical applications, etc. [298, 299]. Advanced forms of micro/nanomotors may accelerate and benefit other research. However, designing and powering micro/nanomotors can be considered as a significant challenge in today’s nanotechnology research. Hence, it is much beneficial for us to learn the state of the art of artificial micro/nanomotors and improve them in this research field. In this section, the published work on the applications of artificial self-propelling micro/nanomotors will be presented and discussed.

**Fig. 14:**
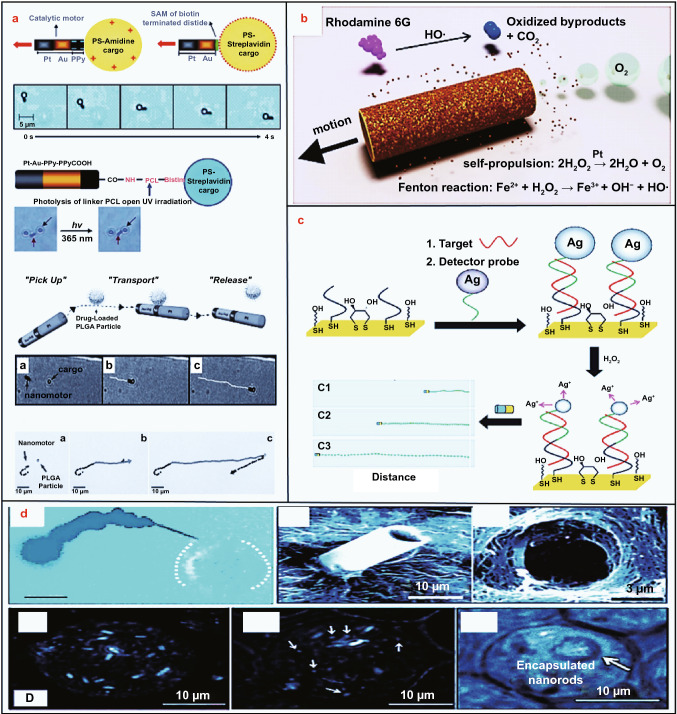
**a** Examples of the delivery of cargo using solid micro/nanomotors: (a) cargo pick-up, (b) cargo delivery, and (c) cargo release, respectively. **b** Organic pollutants degraded by multifunctional micromotors in solutions. **c** Detection of nucleic acid, which alters the propulsion of the micro/nanomotors. **d** A catalytic nanomotor drilling into an immobilized cancer cell

For the cargo delivery by micro/nanomotors, the cargo could simply be attached to the motors by magnetic attraction. The delivery of drug-loaded magnetic poly (d,l-lactic-co-glycolic acid) (PLGA) microparticles has been reported by both chemically propelled and magnetically driven micro/nanomotors, as shown in Fig. 14a [300–302]. For charged cargoes, electrostatic interaction between cargoes and micro/nanomotors could be employed for the pick-up process. A common strategy introducing charged portions into micro/nanomotors is to incorporate a negatively charged polymer part. Sen et al. reported that a polypyrrole (PPy) part was incorporated to a nanowire via electropolymerization, which could be connected to oppositely charged polystyrene amidine cargo via electrostatic interaction, as shown in Fig. 14a. A photo-chemically triggered cargo unloading manner was proposed for cargoes loaded nanowires via electrostatic interaction. An additional Ag portion in a nanowire will be dissolved rapidly in the presence of fuel, chloride ions (Cl^−^), and ultraviolet (UV) light, resulting in drop-off of the cargo. One of the primary environmental applications of micro/nanomotors is to adsorb the pollutants in water. Remediation agents could be incorporated with micro/nanomotors as the outer surface to contribute to the purification process during motion. Soler et al. reported the application of micromotors decorated with an iron (Fe) outer surface to degrade organic contaminants in water via the Fenton oxidation, as shown in Fig. 14b [303]. The application of micro/nanomotors as chemical sensing is based on the case that the motion speed of micro/nanomotors can be converted into an analytically useful signal. The interaction of certain compounds in the sample with the catalytic sites of micro/nanomotors leads to the alteration of their motion speed and is related to the concentration of an analyte in solution, as shown in Fig. 14c [304]. Micro/nanomotors have proven to be able to drill into biomaterials and soft tissues. Rolled-up thin nanomembranes can asymmetrically result in sharp edges being engineered. Micro/nanomotors were self-propelling and externally directed toward immobilized cancer cells as well as embedded in their interior, as shown in Fig. 14d [305]. However, the toxicity of the fuel used for the motion leads to the cells undergoing apoptosis after short periods. Therefore, other environmentally friendly sources of motion are urgently required to be found.

## Conclusions and Outlook

Bioresources are obtainable in large quantities and associated with persistent environmental challenges. It is essential to utilize these resources at a large scale within framework of a circular economy. Furthermore, it is crucial to explore promising technologies for converting bioresource to sustainable energy and materials, which can promote the transformation and application of carbon neutral technology. In the next stage, the renewable energy and materials will be more proactive, to implement new development concepts, support economic green, low-carbon, and high-quality development, and serve the goal of achieving carbon neutrality associated with challenges and opportunities for HALUB. Some future outlooks and prospects are summarized as follows:

1. High-value-added carbon materials production from HALUB

Biomass-modified carbonaceous materials will find relevance for various other carbon-based materials applications like graphene and semi-conductor beyond fertilizer, adsorbent and catalyst. HALUB-derived carbon-based catalysts, including thermocatalysts, electrocatalysts, and photocatalysts are of high interest. Moreover, the high surface area and tunable porosity of the carbon materials can promote the application in carbon capture, such as pressure swing adsorption (PSA). Additionally, the carbon materials have great potentials in lithium battery and hydrogen fuel cell technologies, which play important roles in the green energy for transportation.

(2) Co-conversion technologies

Co-conversion of different HALUB and integration of different technologies are innovative approaches for sustainable bioresource management in the generation of energy and materials. Currently, it is promising to utilize the full components of biomass (cellulose, hemicellulose, and lignin). Combination of biochemical methods (such as AD or fermentation) and thermochemical methods could help to achieve this goal. The components that cannot be converted in biochemical methods (such as lignin) can be used in the thermochemical methods. Actually, lignin is the most abundant aromatic source in the nature.

(3) Application of machine learning in HALUB conversion processes

Due to the difficulty and high cost to perform biomass thermochemical conversion reactions, especially the large-scale reactions. Machine learning plays an imperative protagonist in the optimization of HALUB conversion process and improvement of the conversion efficiency. The current data will help to train the model and thus predict the outcome of the thermochemical conversions. This will rely on a large amount of data and a comprehensive overview of the available experimental results.

(4) Full life cycle assessment of HALUB utilization

HALUB to sustainable energy and material has a positive effect on mitigating climate change and building a community with green future. It is important to emphasize representative feedstocks and utilization approaches in a common framework, including availability, physiochemical characteristics, techno-economic and life cycle considerations for resource-process-use-disposal systems. The utilization of HALUB should be considered in a full life cycle, from cultivation to the final disposal. Wherever possible, use existing indices (e.g., IPCC emission factors) for direct comparison of resource-process impacts.

(5) Future perspectives

The current status and future prospects of HALUB are linked with the maneuver of persistent organic and inorganic pollution and other environmental glitches. Globally, the bioresources management systems can perform a vigorous protagonist in supervision of climate change and subsidiary green revolution. In imminent, renewable materials will be more practical for the implementation of novel developmental perceptions and for achieving carbon neutrality. The promotion of carbon neutral technologies may yield results for conversions of bioresources to biomaterials and energy. There are voluminous opportunities in the field of HALUB based photocatalysis as well as electrocatalysis as it has high curiosity in the field of carbon capture and storing technology to boost modernization. Moreover, hydrogen fuel cell automations perform a vigorous role in green energy. It also promotes scientific knowledge and innovation research hence the green environment will be encouraged. The utilization approaches will enhance and contribute to advancement toward international environments restoration. The unearthing of bioresource derived materials will have momentous impacts on progression of getting environmental sustainability. Although, there are some challenges to comprehend the conversion of energy structure via renewable energy substitutions and need for further development and to contrivance solutions employing HALUB.

In conclusion, this review article explores the utilization of biomass and residual materials associated with persistent environmental challenges around the world and highlights utilization approaches that integrate into circular economy and contribute to progress toward environmental remediation and restoration. Common criticisms of the bioeconomy with regard to challenging HALUB are addressed with feedstocks and utilization approaches in a common framework. At last, challenges and need for further development and to implement solutions utilizing HALUB are discussed.
